# Dynamic Grouping of Hippocampal Neural Activity During Cognitive Control of Two Spatial Frames

**DOI:** 10.1371/journal.pbio.1000403

**Published:** 2010-06-22

**Authors:** Eduard Kelemen, André A. Fenton

**Affiliations:** 1 Department of Physiology and Pharmacology, State University of New York, Downstate Medical Center, Brooklyn, New York, United States of America; 2 The Robert F. Furchgott Center for Neural and Behavioral Science, State University of New York, Downstate Medical Center, Brooklyn, New York, United States of America; 3 Center for Neural Science, New York University, New York, New York, United States of America; Boston University, United States of America

## Abstract

Cognitive control is the ability to coordinate multiple streams of information to prevent confusion and select appropriate behavioral responses, especially when presented with competing alternatives. Despite its theoretical and clinical significance, the neural mechanisms of cognitive control are poorly understood. Using a two-frame place avoidance task and partial hippocampal inactivation, we confirmed that intact hippocampal function is necessary for coordinating two streams of spatial information. Rats were placed on a continuously rotating arena and trained to organize their behavior according to two concurrently relevant spatial frames: one stationary, the other rotating. We then studied how information about locations in these two spatial frames is organized in the action potential discharge of ensembles of hippocampal cells. Both streams of information were represented in neuronal discharge—place cell activity was organized according to both spatial frames, but almost all cells preferentially represented locations in one of the two spatial frames. At any given time, most coactive cells tended to represent locations in the same spatial frame, reducing the risk of interference between the two information streams. An ensemble's preference to represent locations in one or the other spatial frame alternated within a session, but at each moment, location in the more behaviorally relevant spatial frame was more likely to be represented. This discharge organized into transient groups of coactive neurons that fired together within 25 ms to represent locations in the same spatial frame. These findings show that dynamic grouping, the transient coactivation of neural subpopulations that represent the same stream of information, can coordinate representations of concurrent information streams and avoid confusion, demonstrating neural-ensemble correlates of cognitive control in hippocampus.

## Abstract 2

Hippocampal neurons represent two concurrent streams of spatial information by transiently organizing into subpopulations of coactive neurons and can reflect the most behaviorally relevant information at any given time.

## Author Summary

Understanding the world and making optimal decisions requires using the most relevant information while at the same time ignoring irrelevant information, a psychological phenomenon known as “cognitive control.” How the same population of neurons deals with multiple streams of information simultaneously is poorly understood. In this study, we investigated the underlying neural mechanisms of cognitive control in a network of hippocampal neurons known to represent space. We implanted electrodes into the hippocampus of rats and recorded the action potential discharge of many neurons at the same time. The recordings were made while rats that were foraging on a rotating disk used cognitive control to coordinate spatial information from different spatial frames. We found that at each moment, discharge preferentially represented location in one or the other spatial frame. Importantly, we were able to influence the behavioral relevance of these spatial frames, and we found that discharge alternated between signaling location in one or the other frames in accord with its current behavioral importance. The timing of when these neurons were active was also related to their function, such that neurons collectively represented locations in the same spatial frame if they were coactive within a few tens of milliseconds to seconds. We conclude that cognitive control is mediated by a dynamic functional grouping. Neural activity distributed across many neurons transiently organizes into functional groups by coactive firing that represents a coherent stream of information.

## Introduction

At every moment the mammalian brain is confronted with a multitude of sensory stimuli having varying degrees of behavioral relevance and must select from multiple potential responses the ones that are appropriate for the circumstances. The ability to coordinate the processing and use of multiple information streams for selecting an optimal purposeful response has been termed “cognitive control.” It is widely studied, but the underlying neuronal mechanisms responsible for coordination are largely unknown [1].

The processes of cognitive control involve multiple structures within the brain, including the prefrontal cortex [1], the visual [2], and motor systems [3]. Recently, we demonstrated that hippocampus is also involved in cognitive control when a rat has to organize its behavior according to distinct representations of two concurrent frameworks of spatial information [4]. These findings, together with the well-characterized spatial discharge correlates of hippocampal neurons (place cells), put us in a position to investigate the neural mechanisms of cognitive control in hippocampus. To understand how the brain coordinates multiple streams of spatial information, we investigated how two distinct, concurrently relevant spatial representations are coordinated in hippocampal discharge.

Although the hypothesis that information is encoded in the brain by neural discharge patterns that are distributed across a population of neurons [5],[6] has received strong experimental support with the advent of multi-neuron, ensemble-recording techniques in hippocampus [7],[8],[9],[10], the hypothesis encounters a problem when the brain must activate multiple concurrent representations within the same network of neurons. Coactivating more than one distributed representation within the same network causes interference between the different representations and irrecoverable loss of information. Von der Malsburg [11] called this the “superposition catastrophe.” It is analogous to simultaneously displaying multiple messages on the same jumbotron screen. The messages will interfere with each other and become unreadable if they use the same light bulbs at the same time.

The superposition catastrophe could be avoided if different representations were activated at distinct times [11], a process that has been called dynamic grouping [12]. As proposed, dynamic grouping would manifest as a transient “cell assembly,” a coactive subpopulation of neurons whose collective activity encodes a single representation. In the presence of a second concurrent information stream, coactivity within one subpopulation would alternate with the coactivation of another subpopulation that encodes the second representation. Indeed, observations of ensemble discharge that are consistent with dynamic grouping have been reported in rodent hippocampus, where place cell discharge is modulated in time [13] and can switch from one ensemble activity pattern to another in response to misalignments of self-motion and distal cues [14],[15] and for unidentified, possibly internal reasons [8],[16]. However, several studies also did not provide evidence of dynamic grouping when two spatial frames were put into conflict without any explicit behavioral consequences [17],[18],[19],[20],[21]. Thus, while the concept is attractive, it is unclear whether dynamic grouping is a mechanism for coordinating distinct concurrently relevant streams of information in the hippocampus or other networks of the brain.

**Figure 1:**
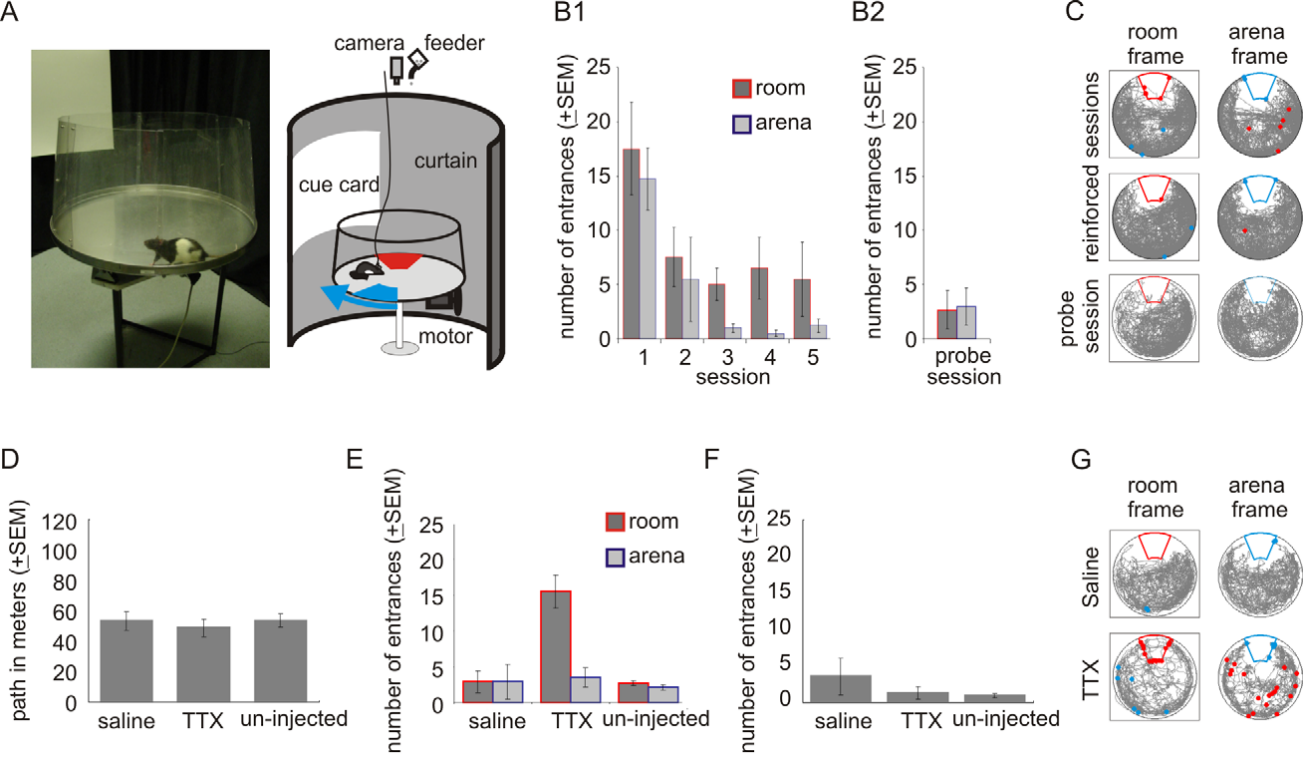
Hippocampus dependent two-frame avoidance. (A) Photograph (left) and schematic (right) of the active place avoidance apparatus. (B1) Learning two-frame avoidance. Entering the shock zones rapidly decreased with training (*n* = 4 rats; F_4,12_≥7.96, *p*s<0.01 for each spatial frame evaluated separately) and was asymptotic after 2 d (sessions 1>2 = 3 = 4 = 5, Newman-Keuls post hoc tests). (B2) Place avoidance was equally good on a no-shock probe session. (C) Example behavior. The rat's path (grey) in the separate room and arena spatial frames and shocks in the room (red) and arena (blue) shock zones are marked by correspondingly colored dots. (D–F) Two-frame avoidance is hippocampus dependent. (D) Injecting TTX or saline into one hippocampus did not affect walking on the rotating arena (F_2,6_ = 0.37, *p* = 0.71). (E) TTX but not saline selectively impaired two-frame avoidance of the room shock zone (F_2,6_ = 9.12, *p*<0.05; Newman-Keuls post hoc tests *p*<0.05*) but not the arena shock zone. (F) TTX did not impair avoidance on the stable arena (F_2,6_ = 1.16, *p* = 0.38). (G) Example of behavior after unilateral saline or TTX injection.

We investigated how concurrent information from two overlapping spatial frames is represented in CA1 principal cell discharge by recording while rats navigated two concurrently relevant spatial frames. A two-frame, active place avoidance task was used. The rat was placed on a slowly rotating disk-shaped arena and trained to avoid two shock zones; one was stationary and the other rotated with the arena [22]. The stationary distal visual landmarks defined the spatial frame of the room while rotating olfactory cues and visual features defined the spatial frame of the arena (Figure 1A; Video S1). We observed that coactive cells tended to represent the same type of spatial information, reducing the risk of interference between the two streams of information. The ensemble preference for one or the other spatial frame changed intermittently within a session; at each moment the more behaviorally relevant spatial frame was more likely to be actively represented in ensemble discharge.

## Results

### Hippocampus-Dependent Coordination of Two Concurrently Relevant Spatial Frames

We first confirmed that rats readily use two concurrent frames of spatial information. The rats rapidly learned the two-frame task after a shaping procedure, which is described in Methods. The average time to enter a shock zone increased from 24.2±3.5 s in the first session of the two-frame task to 380.8±229.5 s by the fifth session when rats only entered a shock zone 5.5±3.4 times during 16 min (Figure 1B). Well-trained rats avoided both shock zones, even in probe sessions when the shock was turned off (Figure 1B2 and 1C). Although the rats never received shocks in the middle of the arena, they did not preferentially spend time there [22]. The rats foraged throughout the arena surface for randomly scattered food pellets but avoided the shock zones (Figure 1C and Figure S1 and Video S1). Note that the room shock zone may have been harder to avoid because the rat is passively brought to it by the arena rotation, while it is never passively brought to the arena shock zone. Thus rats can use and coordinate information from two distinct, concurrently relevant spatial frames [22],[23].

Next we confirmed that this two-frame avoidance depended on hippocampus neural discharge. Injecting the neural activity blocker tetrodotoxin (TTX) into one hippocampus did not alter how much the rats walked (Figure 1D), but it impaired two-frame avoidance (Figure 1E) while sparing avoidance when the arena was stationary (Figure 1F). Specifically, the rats could still avoid the arena shock zone (3.5±1.0 entrances) effectively, but not the room shock zone (15.5±2.4 entrances; t_3_ = 5.48, *p*<0.05). Note that TTX injections into both hippocampi prevent active place avoidance of a single stationary shock zone whether the arena is stationary [24],[25] or rotating [26]. Thus the present result that avoidance of the stationary room shock zone is impaired at the same time that avoidance of the rotating arena shock zone was spared indicates a specific role for hippocampus in coordinating two streams of spatial information beyond its role in spatial memory and navigation, both of which remained intact following the unilateral TTX injection.

These results with avoidance of the two shock zones confirm prior work, in which the ability to coordinate the two spatial frames of information after the TTX injection was investigated in active place avoidance task variants that required the rat to avoid only one shock zone [4],[27],[28]. Those studies showed that the same unilateral TTX injection impaired avoidance of the room shock zone on the rotating arena only when inconsistent room and arena information was concurrently present, but not when the need for coordinating the two spatial frames was removed by stopping the rotation, darkness, or shallow water [4],[27]. All the task variants in this prior work with unilateral TTX injections had a single shock zone and thus equivalent memory load, suggesting that impairment of an ability other than memory itself accounts for the TTX-induced deficit [4],[29]. Consequently, in the present work, although the impairment was only observed in the rotating condition when there were two shock zones and thus greater memory load than when the arena was stationary, we do not interpret the selective TTX effect to be a frank memory deficit. Instead, we conclude that hippocampus is necessary for coordinating information from two concurrent spatial frames [4]. Accordingly, we sought neural correlates of this coordination by investigating CA1 place cell activity during the two-frame task.

### Locations in Both Spatial Frames Are Represented in CA1 Discharge

Putative CA1 pyramidal neurons (*n* = 215) and interneurons (*n* = 9) were recorded from 11 rats. The recordings were made during two-frame avoidance on the rotating arena flanked by two sessions of place avoidance on the stable arena. Ten two-frame sessions were recorded with 10 or more active cells in the ensemble. The spatial properties of pyramidal cell discharge were similar during the stable and rotating conditions (Figure S2 and Table S1; [30]).

**Figure 2:**
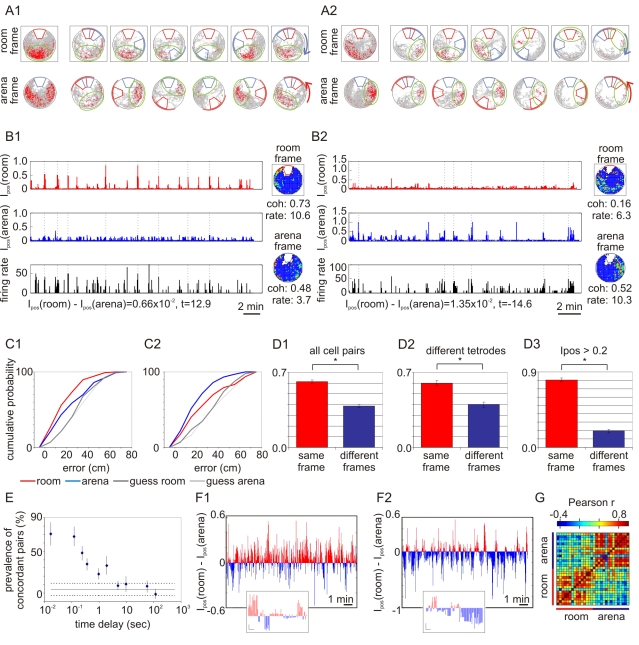
Coordinated frame-specific place cell discharge. (A) Spike maps of a room-preferring cell (A1) and an arena-preferring cell (A2). Frame-specific activity during the whole session is represented in the leftmost column. The other six columns represent activity during different 60° ranges (represented by an arc) of the arena displacement during rotation. (A1) This room-preferring cell fired in the south part of the room (highlighted by the green ellipse) regardless of the arena's orientation in the room. Both measures of spatial firing, average *I_pos_* (0.025) and coherence (0.738), were high in the room frame, but the action potentials were more dispersed across the arena causing lower *I_pos_* (0.009) and coherence (0.484) in the arena frame. (A2) This example of an arena-preferring cell had a firing field with high average *I_pos_* (0.020) and coherence (0.523) in the arena frame. The firing field (highlighted by the green ellipse) was close to the arena shock zone (blue annulus sector). If the rat was willing to visit this region, the cell fired, regardless of the arena's orientation in the room. Firing was dispersed in the room frame, producing low average *I_pos_* (0.006) and coherence (0.162) in this frame. (B) The spatial frame preference was quantified by momentary positional information (*I_pos_*). The time series of *I_pos_* from a single cell in the room frame (red) and *I_pos_* in the arena frame (blue) are shown together with the firing rate (black). The firing rate maps of the cell are shown to the right for both the room and arena frames. The coherence of the rate maps is indicated, as well as the average difference of the *I_pos_* in the two frames. (B1) A room-preferring cell: Instances of high firing rate corresponded to large I_pos_ in the room frame but not large *I_pos_* in the arena frame. (B2) An arena-preferring cell: Instances of increased firing rate were associated with high *I_pos_* in the arena frame but not *I_pos_* in the room frame. (C) Cumulative probability of errors predicting the rat's position in the room and arena frames during two sessions of the two-frame task. Ensemble discharge predicted both room and arena positions better than chance (paired-sample Wilcoxon test C1—room: *N* = 162, *Z* = 6.9, *p* = 10^−11^; arena: *N* = 162, *Z* = 3.9, *p* = 10^−4^; C2—room: *N* = 158, *Z* = 3.0, *p* = 0.003; arena: *N* = 157, *Z* = 7.2, *p* = 10^−12^). While room position was predicted better than arena position in C1 (*N* = 162, *Z* = 3.4, *p*<0.001), C2 demonstrates the opposite (*N* = 157, *Z* = 3.5, *p*<0.0005). (D) Concordant and discordant discharge was examined in pairs of cells that were active in two 117 ms intervals. Concordant and discordant discharge was equiprobable when the intervals were chosen randomly. In contrast, concordant discharge was more likely when cells were coactive in the same interval (D1) even if the cells were recorded from different tetrodes implanted at least 400 µm apart (D2). The proportion of concordant discharge increased when intervals with *I_pos_*>0.2 in one of the frames were analyzed (D3). (E) The frame preference of cell pairs was assessed for cells firing with different time delays from 17 ms to 120 s. The difference between concordant and discordant cell pairs is shown. Horizontal continuous and dotted lines indicate the average and SEM expected differences for independent frame preferences. (F) Ensemble dynamics of frame preference during two-frame avoidance. Ensemble *I_pos_* was computed as the difference between room and arena *I_pos_* during 1 s intervals for each cell summed across all cells in a single recording. Positive values (red) indicate ensemble preference for the room frame; negative values (blue) indicate preference for the arena frame. Insets show segment of each session in finer temporal resolution (scale *I_pos_* difference: 0.1, time: 1 s). (F1) Room *I_pos_* was greater most of the time (65%), indicating a room-preferring ensemble. (F2) Arena *I_pos_* was greater most of the time (70%), indicating an arena-preferring ensemble. (G) Example of a 15-min recording of 14 cells partitioned into separate room-preferring and arena-preferring ensemble spike time series, each composed only of the 117-ms intervals with a reliable corresponding frame preference (|room *I_pos_*−arena *I_pos_*|>0.2). Ensemble activity vectors were computed every minute from each time series and all pairs of vectors were compared and are illustrated as a chronologically ordered correlation matrix. Concordant vector pairs with the same frame preference were substantially more similar (average correlation = 0.55±0.04) than discordant vector pairs with different frame preferences (average correlation = 0.18±0.01).

Location-specific place cell discharge was observed in both spatial frames during two-frame avoidance (Figure 2A, Figure S3). Location-specificity was quantified by *I_pos_*, the positional information in the spike train at each moment. *I_pos_* is always positive as it is an absolute value that derives from our previous work [31]. *I_pos_* was computed separately for the room frame and the arena frame each 117 ms. This interval was chosen because it provided enough time for sampling activity of a putative pyramidal cell, yet it was short enough so that the rat's location did not change much and could be accurately determined [31]. Moments when *I_pos_* was large in one frame and low in the other frame suggest that the cell's momentary discharge preferentially represents a location in the frame with higher *I_pos_* (Figure 2B).

We judged a cell to prefer firing in one spatial frame if the average *I_pos_* from the entire session was greater in one frame according to a Wilcoxon signed rank test. The majority (75.4%) of place cells had a statistically significant preference for location-specific discharge in one of the frames during a session of two-frame avoidance. While 52.5% of place cells had a room frame preference (Figure 2A1, 2B1), a similar proportion (47.5%) had an arena frame preference (Figure 2A2, 2B2; test of proportions: *z* = 0.67, *p* = 0.5). Consistent with previous reports that distal as well as local stimuli can control place cell spatial firing [17],[19],[30], we found that CA1 discharge signaled locations in both spatial frames during two-frame avoidance.

### Locations in Both Spatial Frames Are Represented in Ensemble Discharge

We next used position reconstruction [7],[9],[13] to investigate whether the rat's position in the two frames was represented in CA1 ensemble firing. Ensemble discharge predicted the position of the rat in each frame significantly better than chance in 9 of the 10 recordings we examined (see examples in Figure 2C). In 7 out of 10 recordings, the position in one of the spatial frames was more accurately reconstructed (Figure 2C), suggesting a spatial frame preference of the recorded ensembles.

Ensemble *I_pos_* was related to the quality of reconstructing the rat's position. Location in the arena was reconstructed better when *I_pos_* in the arena frame was high (t_9_ = 4.62; *p*<0.005). Similarly, room location was predicted better when *I_pos_* was high in the room frame (t_9_ = 3.66; *p*<0.01; Figure S4). We conclude that locations in both spatial frames were represented during two-frame avoidance.

### Coordinated Representations of the Two Spatial Frames

It has been hypothesized that dynamic grouping may be a mechanism for coordinating different concurrent representations by activating them at distinct times [11],[12]. We sought evidence of dynamic grouping during two-frame avoidance by analyzing the ensemble dynamics on shorter timescales, ranging from tens of milliseconds to minutes. Analysis of ensemble *I_pos_* revealed that neurons that are coactive tend to represent locations in the same spatial frame. If a cell pair discharged within the same 117 ms interval, a concordant frame preference was more likely (Figure 2D1) and greater than for randomly selected intervals (t_36_ = 6.2; *p*<0.01). Coactive cell pairs recorded from locations at least 400 µm apart had the same tendency for concordant responses (Figure 2D2).

Although concordant responses predominated, a substantial number of coactive cells also had discordant frame preferences (Figure 2D1,2). We considered the possibility that imperfect assignment of a cell's activity to one or the other frame caused truly concordant frame preferences to be misclassified as discordant. The analysis was repeated on only the moments when a frame preference was especially strong, and therefore more likely to be correct. For this purpose only intervals when *I_pos_* in at least one frame was higher than 0.2 bits for both cells were analyzed. Just 4.4% of the original dataset met this criterion. The proportion of concordant cell pairs in this subset of data was 79.8%±2.9% (Figure 2D3), substantially higher than when the less strict criterion was used (t_27_ = 6.1; *p*<0.001). We also analyzed intervals when the difference between room and arena *I_pos_* was greater than 0.1 bits, on the assumption that this selected only those intervals with a strong frame preference. Only 7.9% of the data met this criterion, and the proportion of concordant cell pairs in this subsample was 80.8%±2.6%, again much higher than when the less strict criterion was used (t_27_ = 6.7; *p*<0.001). Together these results indicate a strong tendency for coactive cells to signal location in the same spatial frame.

Next we estimated the duration of this concordant frame-preference in CA1 discharge by repeating the analysis for a range of time delays between 17 ms and 2 min (Figure 2E). The tendency for concordant responses was strongest when cells fired together within the shorter time intervals. The preference for concordant discharge was most prominent at sub-second intervals and persisted for a couple of seconds, confirming that signaling of locations in the same spatial frame was transient, as predicted by dynamic grouping.

Further analysis of the time series of ensemble *I_pos_* revealed that the assembly of CA1 neurons into a same-function group of coactive cells was transient because the assemblies alternated between representing room and arena locations. We studied the time series of differences between ensemble *I_pos_* in the room frame and arena frames (e.g., Figure 2F, Figure S9). Episodes associated with a preponderance of room frame information were interleaved with episodes with a preponderance of arena frame information. If the ensemble as a whole was changing the frame preference with time, then it should be possible to predict the momentary frame preference of a particular cell from the known frame preference of the rest of the ensemble. In a leave-one-out analysis we computed ensemble I_pos_ from all cells in the ensemble except one. When the rat moved through the excluded cell's firing field, *I_pos_* was large when the cell was active [31]. During these intervals of activity the correlation between the ensemble and individual cell's *I_pos_* values was typically significantly greater than zero (r = 0.31±0.01; t_122_ = 17.9, *p* = 10^−34^). When the absolute difference between *I_pos_* in the two frames was high (>0.2 bits) and more reliable, the correlation was even stronger (*r* = 0.47±0.03; t_49_ = 9.9, *p* = 10^−13^). These observations reveal that the ensemble, not just single cells, alternated frame preference during two-frame avoidance. Indeed, in every recording, the activity patterns across the ensemble were more similar for intervals with the same frame preference than different frame preferences (Figure 2G). Analysis was performed for each recording separately and the difference was significant in 7 of the 10 recording sessions.

### Representations of Locations in One of the Two Spatial Frames Dominate Each Session

Location-specific information from a single spatial frame dominated CA1 ensemble discharge in single recordings even though discharge fluctuated between representing locations in the two spatial frames. The dominant frame during a single session could be the room frame (Figure 2F1) or the arena frame (Figure 2F2). In the 10 sessions with at least 10 cells, ensemble activity during a single session represented the dominant spatial frame 60.1%±1.9% of the time. According to the test of proportions, 9/10 recordings had a significant predominance for a spatial frame during the session (*p*s<0.05). Similarly, all recordings had a significant frame predominance when room frame and arena frame *I_pos_* were compared by Wilcoxon signed rank test (*p*s<0.05).

**Figure 3:**
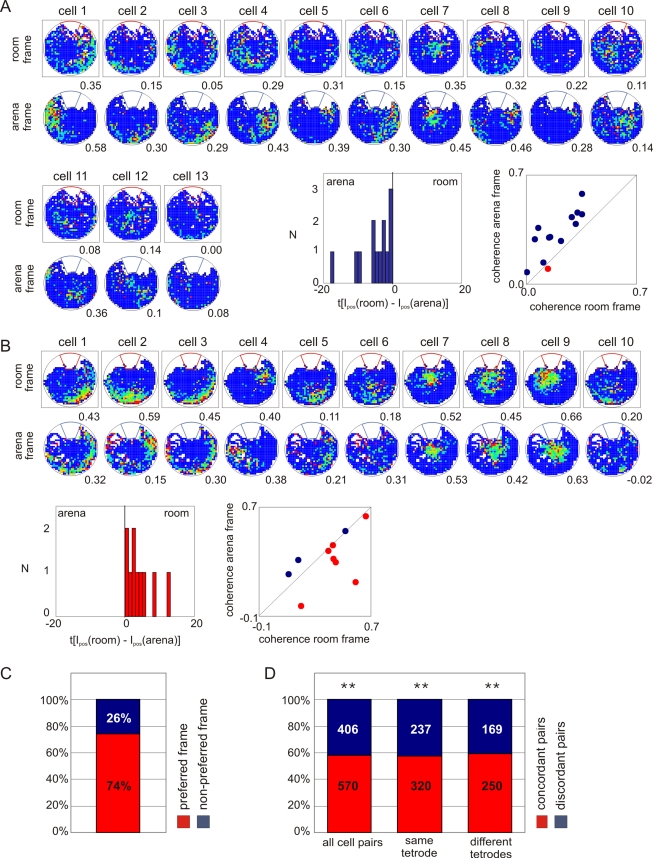
The ensemble preference for one spatial frame dominates two-frame avoidance sessions. An example of an arena-preferring (A) and a room-preferring (B) ensemble. The difference between room frame and arena frame *I_pos_* for each cell indicates an arena preference in Ensemble A and a room preference in Ensemble B. Scatter plot of arena map coherence and room map coherence also indicates the ensemble frame preferences. (C) Most cells had a firing field in the ensemble-preferred frame. (D) Simultaneously recorded cell pairs were more likely to have a concordant frame preference than a discordant frame preference. The tendency for an excess of concordant cell pairs was observed when all cell pairs (*z* = 5.24; **p<0.0001), cell pairs from the same tetrode (*z* = 3.52; **p<0.0005), or different tetrodes (*z* = 3.96; **p<0.0001) were analyzed (χ^2^<0.15, *df* = 1, *p*>0.5).

The predominance of ensemble discharge to signal locations in the same spatial frame was confirmed using location-specificity measures that average discharge of a single cell across the entire recording, which is the conventional way of analyzing place cell data. Analysis of the firing rate map coherence confirmed that most cells in a recording had their firing better organized in the same frame (Figures 3A–3C, S5A). This observation was quantified by classifying pairs of simultaneously recorded cells as “concordant” if they both had the same frame preference or “discordant” if they had different frame preferences. More concordant cell pairs were observed than expected by chance. The predominance of concordant frame-specific discharge in cells recorded within the same session was robust. It was independent of the parameter used to assess the spatial organization of firing; both average *I_pos_* (test of proportions: *z* = 5.25, *p*<0.0001; Figure 3D) and coherence (test of proportions: *z* = 3.71, *p*<0.0005, Figure S5A) showed significant predominance of concordant cell pairs. The results were the same whether all active cells or active cells with statistically significant frame preference (test of proportions: *z* = 4.13, *p*<0.0005) were analyzed (Figure S5B). This tendency for an ensemble frame preference was equally strong when we only evaluated anatomically nearby or anatomically distant cell pairs recorded from the same or different tetrodes, respectively (Figure 3D, Figure S5).

Session-averaged spatial frame preferences did not correlate with session-averaged behavior (Figure S10).

The frame preference of cells and ensembles was not hard-wired because the preferences could change between recording sessions. The same ensembles were recorded from a rat trained to do two-frame avoidance in two different rooms. Sometimes the ensemble frame preference differed in the two rooms (Figure S6). These analyses show that on the timescale of several minutes, the discharge of the CA1 population is grouped according to their presumed function—encoding locations in a specific spatial frame but that the frame preference changes between sessions.

### Neural Synchrony within Same-Function Neural Groups

**Figure 4:**
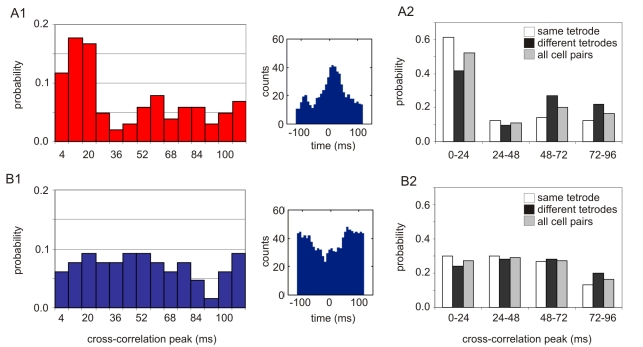
Neural synchrony in concordant cell pairs. (A1) Concordant cell pairs preferentially discharge together within 24 ms (102 cell pairs: χ^2^ = 44.31; *df* = 13; *p* = 10^−5^). Right: A spike train cross-correlogram for a concordant cell pair. (A2) The same tendency was observed whether cell pairs from the same (χ^2^ = 34.35; *df* = 3; *p* = 10^−7^) or different tetrodes (χ^2^ = 8.46; *df* = 3; *p* = 0.04) were analyzed. (B1) Discordant cell pairs discharge independently in time (65 cell pairs: χ^ 2^ = 5.43; *df* = 13; *p* = 0.97). Right: A spike train cross-correlogram for a discordant cell pair. (B2) The tendency is the same for cell pairs from the same (χ^2^ = 2.27; *df* = 3; *p* = 0.5) or different tetrodes (χ^2^ = 0.44; *df* = 3; *p* = 0.9).

The preference of coactive cell discharge for locations in the same spatial frame was most prominent at sub-second intervals so we investigated whether synchronous neuronal discharge might also contribute to coordinating the two spatial frames of information. Spike train cross-correlations were analyzed if the cell pair was coactive within 128 ms at least 100 times. This means that cells with overlapping firing fields were analyzed. The firing fields could overlap whether they were organized in the same frame or in different frames, when the room and the arena were aligned appropriately. As reported for linear tracks [32],[33], the time of the cross-correlation peak was related to the similarity of the two firing fields (see Text S1). Concordant cell pairs (with the same frame preference as measured by average *I_pos_*) were more abundant (102 pairs) than discordant cell pairs (65 pairs; test of proportions: *z* = 2.2; *p* = 0.01), consistent with the predominance of concordant hippocampal firing. The concordant cell pairs had an increased tendency to discharge together within 25 ms (χ^2^ = 44.31; *df* = 13; *p*<0.0001; Figure 4A). In contrast, discordant cell pairs had no tendency to fire together (χ^2^ = 5.43; *df* = 13; *p*>0.9; Figure 4B). These results were also confirmed when the cells from different tetrodes implanted at least 400 µm apart were analyzed (Figure 4A2, 4B2). The prevalence of coactivity within ∼25 ms in concordant cell pairs supports the hypothesis that dynamic grouping [34] may contribute to coordinating hippocampal representations of space by defining cell assemblies, same-function subpopulations of coactive neurons [8].

### Neural Ensemble Correlates of Two-Frame Avoidance Behavior

A picture emerges from these data where a distributed subpopulation of cells discharges together, coalescing into a group that collectively represents locations in the same spatial frame, and activation of these functionally defined groups alternates. Next we analyzed if the dynamically changing ensemble activity pattern is related to the changing behavioral significance of the two spatial frames.

**Figure 5:**
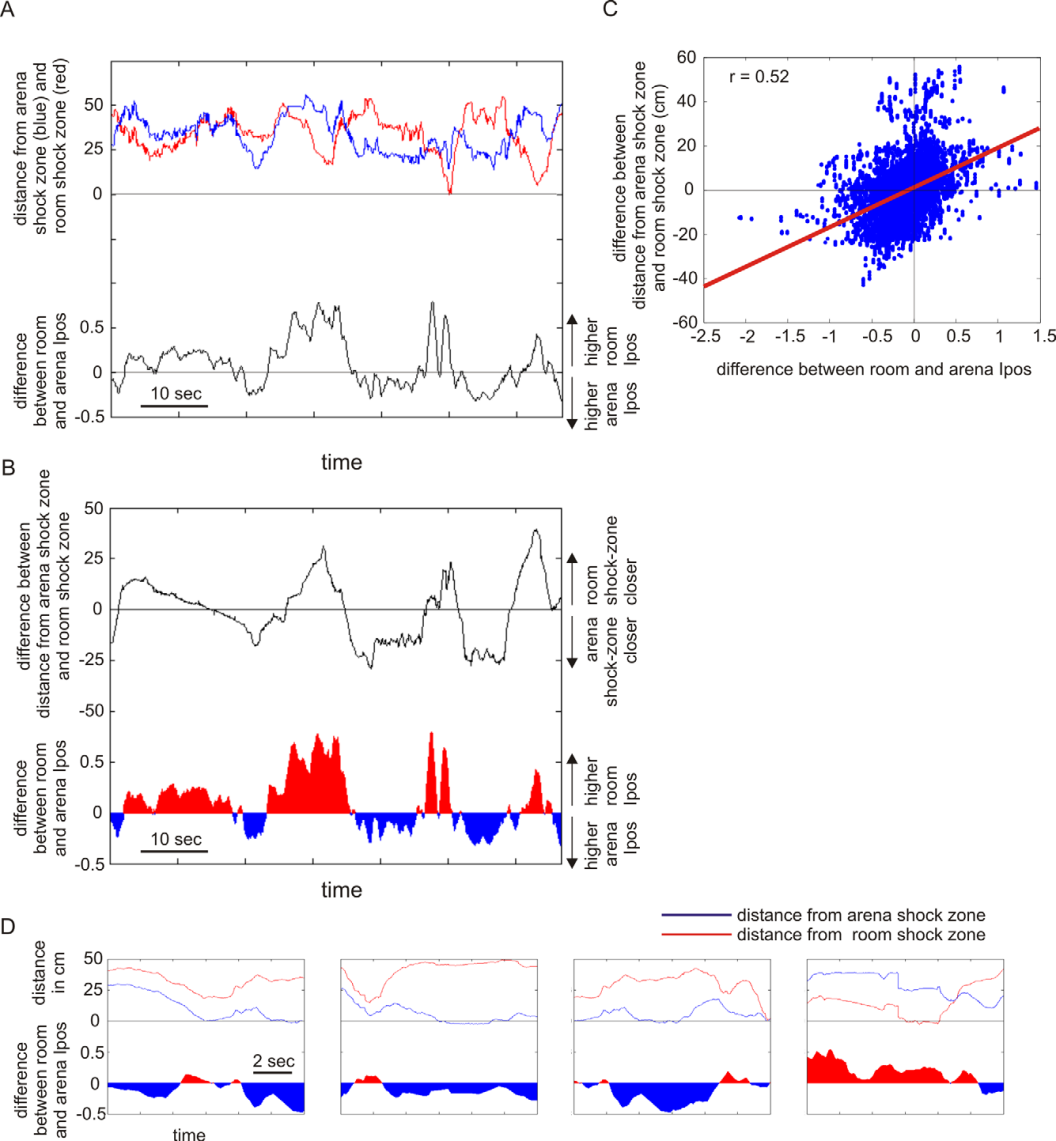
Ongoing frame-specific ensemble discharge is related to concurrent two-frame behavior. (A) The distances of the rat from the room (red) and arena (blue) shock zones and the difference between room and arena ensemble *I_pos_* (black) are shown for 80 s. Ensemble activity better indicated locations in the same spatial frame as the nearby shock zone. (B) The difference between distances from the two shock zones (black line) and the difference between the ensemble *I_pos_* in the two spatial frames covary. (C) This correspondence was observed during the entire session. (D) Ensemble activity during the 5 s before and after entering a shock zone (*y*-axis labels as in panel B). These examples from a session with the shock off illustrate that ensemble activity typically reflects the “riskier” frame that defined the nearby shock zone and that the rat did not go deep into a shock zone.

When a rat was closer to the arena shock zone than the room shock zone, ongoing ensemble activity indicated the rat's arena position better than its room position, and when the rat was closer to the room shock zone the ensemble preference reversed to indicate the room location better than the arena location (Figure 5). Furthermore, analysis of frame-specific positional information at the moments the rat entered the shock zones revealed an even stronger relationship between neural discharge and avoidance behavior. Ten of 14 entrances into the arena shock zone were associated with greater arena frame ensemble *I_pos_* and 8 of 10 entrances into the room shock zone were associated with greater room frame ensemble *I_pos_*. This pattern of entering the better-represented shock zone was unlikely to occur by chance (18/24 versus 50% chance; *z* = 2.45; *p* = 0.007), suggesting that CA1 neuronal discharge was tuned to represent the more relevant, “riskier” spatial frame as the rat approached and entered a shock zone. It is tempting to speculate that shock-zone entrances could be a consequence of the rat's deliberate investigation of the shock zone, rather than miscalculations. When a rat entered the better-represented shock zone location, the ensemble frame preference persisted in the same frame as the shock zone for 1.8±0.5 s. In contrast, the ensemble frame preference only persisted for 0.2±0.1 s, when the rat entered a shock zone that was inconsistent with the concurrent ensemble frame preference (t_14_ = 2.51; *p*<0.05).

This coherent relationship between the behavioral significance of the two spatial frames and the ensemble preference for representing locations in the frame that is more behaviorally significant indicates a strong link between the rat's momentary behavior and the information being signaled by concurrent hippocampal ensemble discharge. These data strongly indicate that the dynamic grouping of hippocampal ensemble activity we observed during two-frame avoidance is a correlate of cognitive control in the two-frame task.

## Discussion

### A Neural Ensemble Activity Correlate of Cognitive Control

While rats demonstrated cognitive control in a two-frame avoidance task, we identified that the collective activity within coactive subpopulations of neurons alternated between signaling locations in one then the other of the two spatial frames, directly demonstrating that this dynamic grouping is an ensemble activity correlate of cognitive control in hippocampus. We found that at each moment the discharge of the active subpopulation of anatomically distributed cells preferentially represented the rat's location in one of the two spatial frames. Thus the coactive subset of CA1 neurons defined a neural group with the common function of representing location in a single spatial frame. This functional grouping was transient because the ensemble neural activity toggled between representations of the two spatial frames.

The tendency of coactive neurons to represent location in the same spatial frame was observed at multiple timescales that ranged from minutes to milliseconds. The dynamic organization appeared as a predominance of information about locations in one spatial frame during 15-min recordings (Figure 2F, Figure 3). The same tendency also occurred at shorter timescales. Cells would coactivate within ∼2 s if they represented nearby locations in the same spatial frame (Figure 2E) and within ∼25 ms if they represented the same location in a single spatial frame (Figure 4A), consistent with the binding by synchrony [35] and cell assembly hypotheses [5],[8] and attention-like modulation of hippocampal place cell activity [36]. The dynamic functional organization of ensemble responses mirrored the rat's two-frame avoidance behavior. There was a strong correspondence between the spatial frame of the shock zone that the rat was approaching and the spatial frame of the location being represented in CA1 discharge (Figure 5). Thus ensemble neural discharge in hippocampus correlated with the ability of the rat to process information in two concurrent spatial frames without confusion, and also with the ability to coordinate the activation of one spatial information stream over the other in accord with optimal ongoing avoidance behavior. The dynamic functional grouping of ensemble neural activity in the hippocampus we observed is thus a correlate of cognitive control.

### A Role for Hippocampus in Cognitive Control

While the mechanisms of cognitive control that coordinate distinct conflicting representations have been investigated in various primate brain systems [2],[3], most commonly in frontal cortex [1], accumulating evidence suggests that the rodent hippocampus also provides a powerful model for elucidating mechanisms of cognitive control. The present study follows directly from recent work indicating a critical role of hippocampus in coordinating distinct streams of spatial information [4],[27],[29] that extended beyond the well-established roles of hippocampus in memory storage [37],[38],[39],[40] and spatial navigation [41]. Bilateral TTX injections into dorsal hippocampus impaired active place avoidance variants regardless of whether the arena was stable or rotating, confirming the well-established role of hippocampus in spatial memory and navigation.

In contrast, memory and navigation were spared when TTX was injected into only one dorsal hippocampus because the injection only impaired those active place avoidance task variants that required coordination of information from two spatial frames [4],[28]. In the experiments that observed an effect of the unilateral TTX injection, a rat on the rotating arena had to avoid the room shock zone and ignore the task-irrelevant location of shocks in the spatial frame of the rotating arena. Note that the same injection was not impairing in very similar control task variants when information in the interfering spatial frame was hidden. This was achieved either by turning off the lights to make room cues invisible or by covering the arena surface with shallow water to hide the local olfactory cues. The present study confirmed and extended this prior work by demonstrating that an intact hippocampus is required for coordinating information from the two dissociated spatial frames at the same time that spatial memory and navigation were intact (Figure 1E).

Coordinating interactions have been described in the action potentials and local field potentials of hippocampus and prefrontal cortex [42],[43]. We may thus speculate that the hippocampus is a component, perhaps a key component, of the network of structures important for cognitive control. The selective failure of rats to coordinate avoidance of shock zones in two spatial frames after local hippocampal infusions of TTX and the demonstration of dynamic functional grouping in CA1 discharge provide two complementary lines of evidence for a key hippocampal role in cognitive control. Although cognitive control failures are central to schizophrenia [44] the pathophysiology of the failure has not been investigated at the level of neuronal network discharge [45],[46]. That may soon change now that a robust neural ensemble correlate of this mental ability has been identified in the rat.

### Dynamic Organization of Hippocampal Activity

The present findings extend prior work demonstrating that hippocampal activity is dynamically organized on a sub-second timescale. We previously reported an extreme variability in place cell firing [13], and theoretical work suggested that this overdispersion of place cell discharge could arise from a dynamic attention-like modulation of firing on the timescale of about a second [47]. Extending these results, distinct states were identified in hippocampal ensemble activity and attributed to spontaneous switching between multiple hippocampal maps [16],[36]. In the present work, for the first time, CA1 ensemble activity was studied in a situation where the rat had to organize its behavior according to two distinct concurrently relevant spatial frames. We demonstrated a direct relationship between the dynamic organization of CA1 ensemble discharge and the cognitive control of purposeful behavior.

### Prior Studies on Hippocampal Representations of Distinct Spatial Frames

Our work builds on previous efforts to study hippocampus place cell discharge in response to distinct, conflicting sets of landmarks [14],[15],[17],[18],[19],[48],[49],[50]. Our approach advanced the previous efforts in several ways. Most prior reports averaged the activity of cells over the entire session. While this was in accord with the standards of place cell research, it precludes observing any of the short timescale dynamic organization of hippocampal discharge that appears to be critical for hippocampal function, and cognitive control in particular. Furthermore, rats in our two-frame task were directly reinforced to use information from both sets of landmarks more or less concurrently. In other words, the explicit demand for cognitive control was built into the task in steady-state conditions rather than relying on perturbation, probe trials, as was done in prior work. Our approach ensured that information about both frames was being processed and used during the recordings. It also allowed direct investigation of the relationships between the rat's purposeful behavior and hippocampal activity. A more extensive discussion of previous work can be found in Text S1.

### Summary

In summary, we recorded CA1 place cells as rats performed a two-frame task that requires cognitive control to coordinate the use of information from two concurrent spatial frames. In accord with previous work, coordinating the two streams of spatial information required an intact hippocampus. We observed a robust dynamic grouping of CA1 activity into coalitions of coactive cells that processed spatial information from the same frame. Cells that were coactive within 2 s or less were preferentially responding to locations in the same spatial frame, and the momentary frame preference of the ensembles alternated each few seconds throughout the sessions. The ensemble neural activity at any instant reflected the relative behavioral relevance of the two spatial frames at that time. This collection of findings indicates that the dynamic grouping of ensemble discharge is a neural correlate of cognitive control in hippocampus.

## Methods

All experimental procedures have been previously published in detail, complied with NIH and institutional guidelines, and were approved by Downstate Medical Center's Institutional Animal Care and Use Committee.

### Place Avoidance

The rat was placed on a metal disk-shaped arena (82 cm in diameter) that contained olfactory, tactile, and visual landmarks. A transparent wall (40 cm high) prevented the rat from jumping off the disk. The arena was situated in an experimental room and surrounded by a black curtain with a white polarizing card. The arena was stationary for stable sessions and it rotated continuously at 1 rpm during rotating sessions. At each instant the rat's position could be defined relative to the spatial frame of the room, and relative to the spatial frame of the arena. Multiple landmarks could be used to recognize the rat's position in the room: a white card on the curtain, the entrance through the curtain, and other visual and auditory cues. The position in the arena could be recognized by tactile and olfactory landmarks on the arena surface as well as by visual landmarks (strips of tape) on the transparent wall [4].

The rat was reinforced to avoid two shock zones. A computer-controlled experimental system was used. Every 17 ms the system tracked the rat's position in the room and its position on the arena from the output of an overhead video camera (Bio-Signal Group Corp., Brooklyn, New York). The room shock zone was defined relative to the room landmarks, and the arena shock zone was defined relative to the arena landmarks (see Figure 1 and Video S1). Each shock zone was an annulus sector that spanned 45° and extended across the outer 60% of the arena radius [22]. When the rat entered a shock zone a mild constant current (60 Hz, 0.3 mA) foot shock was delivered for 500 ms. In order to reinforce the rats to walk on the arena, they were food deprived and trained to forage for 20 mg food pellets that where scattered at random locations of the arena from an overhead feeder at 20 s intervals.

The position of the rat and the displacement of the arena within the room were monitored by tracking two infrared light emitting diodes (LED) attached to the rat and to the arena, respectively. The rat's LED was connected to the unity-gain buffering headstage amplifier. The arena LED protruded 19 cm from the edge of the arena below the disk surface. The position of the two LEDs was tracked from an overhead video camera by commercial software running in a computer in the adjacent room. The software tracked the rat's position in the room and on the arena; it also controlled the delivery of shocks and food pellets and stored the behavioral data for offline analysis.

### Behavioral Training

The rats were first food deprived to 90% of their free-feeding weight. Then they were trained to do the Room+Arena+ two-frame active place avoidance task using a shaping procedure of three phases. In the first phase of shaping, the rat was trained to forage for food pellets that were randomly scattered on the arena. The arena was stationary during this phase and the shock was off. The first phase of training lasted two to five 20-min sessions until the rat would spend most of the session actively foraging on the arena. During the second phase of training, the rat was trained to do the (Room&Arena)+, one-frame task variant. The rat was reinforced to avoid a shock zone while the rat was foraging on the stationary arena. This phase of training lasted two to five sessions until the rat learned to forage continuously and avoid the shock zone. During the third, final stage of training, the rat learned the Room+Arena+ task variant. During this stage, each session of the (Room&Arena)+ control task was followed by a session of the Room+Arena+ task variant on the rotating arena. The foraging rat was reinforced to avoid both the room shock zone and the arena shock zone. The rats were exposed to one or two training sessions per day.

### Intrahippocampal Injections

Five rats were trained until performance was asymptotic in the place avoidance tasks with the arena stable (10 min) and rotating (15 min) before undergoing surgery to implant a pair of guide cannulae with the tip 3 mm above the injection target in the dorsal hippocampus (relative to Bregma AP 3.5 mm; L 2.5 mm; DV 4 mm). The detailed procedure for implanting and subsequently injecting the rats has been published [4],[51]. A week after surgery the rats were retrained to reestablish optimal place avoidance performance. One rat could not regain the pre-surgical level of performance and was excluded from further study. Before testing the effect of the TTX injection on place avoidance, the rats received a bilateral injection of TTX (5 ng/µl/side) and were left in the home cage to habituate to the procedure.

A within-subjects design was used to test the effect of injecting TTX into one hippocampus. Each rat was injected and allowed to rest in the home cage until training in the familiar task began 1 h later. The injection infused either saline or TTX into the left or right hippocampus. Performance after injections was compared to sessions in which no injection was given. Each rat received the four possible injections and the session without injection in a counterbalanced order. There was an interval of at least 1 d between injections. The performance of each rat after saline and TTX injection was taken as the average of the corresponding pair of sessions. These average data were compared by one-way ANOVA with repeated measures to test for the effect of treatment (no injection, saline, TTX) and Newman-Keuls post hoc comparisons were performed.

### Electrode Implantation

Rats were trained in the place avoidance tasks prior to the implantation of 25 µm nichrome wire electrodes. Under pentobarbital (50 mg/kg i.p.) anesthesia, eight independently movable tetrodes were implanted above the hippocampal CA1 subfield (2.5 mm lateral and 3.8 mm posterior from Bregma) and cemented to the skull. After a week of recovery the rats were retrained, and the tetrodes were gradually advanced into the pyramidal layer of CA1, until hippocampal complex spike cells could be recorded. The signal was amplified 5,000–10,000 times, filtered between 500 Hz and 5 kHz, and digitized at 32 kHz or 48 kHz, using custom (AcX, A.A. Fenton) or commercial software (dacqUSB, Axona Ltd.). Action potential waveform (2 ms duration) data were stored and analyzed offline. Single unit discrimination was done using custom software (Wclust, A.A. Fenton) to identify clusters of events in a multi-dimensional waveform parameter space (Figures S7, S8).

### Quality of Spatial Discharge

Frame-specific spike maps and session-averaged spatial firing rate maps were computed for each single unit. These spatial representations of action potential discharge were computed for both the room spatial frame and the arena spatial frame. In each spike map, gray lines represent the rat's track and the location of the rat when a cell discharged is represented by a red dot. The spatial resolution of the spike maps was 0.3 cm. The pixel resolution was 2.5 cm for firing rate maps. The rate in each pixel was computed as the total spikes that were discharged when the rat was in the pixel divided by the total time the rat was in the pixel. The rate map coherence [52] and momentary positional information [31] were used to quantify the spatial organization of a single cell's discharge.

Spatial coherence characterizes the similarity in the firing rate between adjacent pixels of a firing rate map. The firing rate in each pixel was compared with the average firing rate of the eight adjacent pixels. The average firing rate of the eight pixels was computed by dividing the spike count in the eight pixels by the time spent in the eight pixels. The Pearson correlation between the original firing rate in each pixel and the average of the eight neighbors was computed. The correlation coefficient (*r*) was *z*-transformed to compute coherence. Spatial coherence relies on averaging location-specific spiking across long periods of time, typically the entire recording; consequently the measure is quite insensitive to short-timescale changes in spatial tuning.

We aimed to evaluate whether the spatial tuning of place cell discharge changed from one spatial frame to another on short timescales, which is why we sought a measure that estimates how much the discharge during a short time interval signals about the rat's location at that moment. We therefore adapted a measure we call momentary positional information (*I_pos_*) from the previously introduced measure “positional information” [31] (Figure S11). The relationship between the two measures is described in Text S1.

Momentary positional information–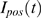 can characterize the location-specific firing of a cell during a brief time interval (Δt = 117 ms). It is defined as:
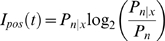
(1)


*P_n_* is the unconditional probability of the cell firing *n* spikes during the interval; *P_n|x_* is the conditional probability of firing *n* spikes if the rat is in location *x* during the interval. 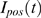 has units of bits and is zero if the discharge observed in a location has the same overall probability (independent of location) as the probability in the current location. 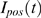 can be positive or negative. However, the absolute value of 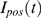 is large whenever the number of spikes observed at the location is distinct or “surprising” compared to the unconditional probability of observing the same number of spikes. We denote |
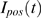
| using the shorthand, *I_pos_*. A cell's spatial frame preference was determined by first calculating *I_pos_* separately for each spatial frame. The preferred frame was the one with the larger average *I_pos_*. The statistical significance of the frame preference was determined by comparing the two frame-specific distributions of *I_pos_* by the non-parametric paired-sample Wilcoxon signed rank test.

A time series of *I_pos_* values for two hippocampal place cells recorded during the two-frame avoidance on the rotating arena is shown in Figure 2. The two time series are plotted together with the momentary firing rates of the cells. The figure shows that at moments of increased firing rate, the values of momentary positional information increase in one reference frame but not in the other. This indicates that the cell's firing preferentially signaled information about the rat's position in one reference frame but not the other.

### Ensemble *I_pos_*

Just as the time series of frame-specific *I_pos_* can characterize the temporal dynamics of location-specific discharge in the activity of a single cell, the aggregate *I_pos_* in an ensemble of cells can characterize the temporal dynamics of ensemble discharge at each short moment of a recording. We defined ensemble *I_pos_* as the sum of *I_pos_* across all the cells in an ensemble. We computed ensemble *I_pos_* for each spatial frame at every 117 ms time step.

### Reconstruction of the Rat's Path

We used a template-matching position reconstruction algorithm [7],[9],[13] to predict the rat's room and arena position from ensemble discharge during each 2-s interval. The activity of simultaneously recorded cells was characterized as a population firing rate vector at each time step. At each location the average firing rate of each cell in the ensemble contributed to a location-specific template firing rate vector. The decoded position was the location with the maximal projection of the current firing rate vector onto one of the location-specific template vectors. If there was no activity during a time step, no position was decoded for the time step. The location-specific template vectors were computed from all but the last 5 min of each recording. The remaining data were used for decoding position from ensemble spiking. To estimate chance the reconstructed location at each time step was chosen by chance from amongst the visited locations. This method is a simple, explicit test of how well the rat's location is predicted by location-specific firing rate itself, without assumptions about the dynamics of the discharge. The accuracy of prediction was similar to values reported previously [7].

### Correlation Matrix of Ensemble Activity

The ensemble frame preference was studied with a time resolution of 117 ms. The 117 ms intervals were judged as room or arena preferring based on ensemble *I_pos_*. Only intervals with |room *I_pos_*–arena *I_pos_*|>0.2 were further analyzed. For each 1-min period of a recording, the room-preferring intervals were grouped together, and arena preferring intervals were grouped together, and the mean firing rate of each cell was computed. Thus for each minute of the recording we computed an average ensemble activity vector for room-preferring intervals and an average ensemble activity vector for arena-preferring intervals. For a 15-min recording this would produce 15 room vectors and 15 arena vectors. Each pair of vectors was then compared by Pearson correlation, and the results are shown in the form of correlation matrix (Figure 2G). The lower left-hand quadrant compares room-preferring ensemble vectors during 1-min periods of the session. The upper right-hand quadrant compares arena-preferring ensemble vectors during 1-min periods of the session. The lower right-hand quadrant compares room-preferring ensemble vectors with arena-preferring ensemble vectors. Because there are different cells in each recording, this analysis must be performed separately for each recording session.

### Cross-Correlation Analysis of Spike Timing

Cross-correlation histograms were computed for all simultaneously recorded cell pairs. Cell pairs were only studied further if they were coactive enough to have discharged at least 100 action potentials within 128 ms of each other. The histograms were computed at 4 ms resolution. The peak of the histogram between 0 and 128 ms was identified after smoothing by a 12 ms running average. This peak was taken as the preferred temporal offset for the cell pair's discharge. The distribution of the histogram peaks was compared against a homogeneous expectation by chi-square statistics.

Cell pairs with overlapping firing fields that fire closer in time tend to have more similar firing fields. On linear tracks, two cells will discharge closer in time if their firing field centers are closer in space [32],[33]. We observed a similar relationship during two-frame avoidance in cell pairs with the same frame preference (*r* = −0.37, *p* = 0.001) as well as for cell pairs with opposite frame preferences (*r* = −0.30, *p* = 0.03) and also when all the cell pairs were analyzed together (*r* = −0.37, *p*<0.001). As expected, the similarity of firing fields was greater for concordant cell pairs than for discordant pairs (t_123_ = 2.6, *p* = 0.01). However, there was no difference in the number of counts in the cross-correlograms of concordant and discordant cell pairs (t_123_ = −0.34, *p* = 0.74).

## Supporting Information

Figure S1 **Time spent in different parts of the arena and room during three different sessions of the two-frame task.** The time spent in each pixel is color coded. The dwell time in seconds of the most visited pixel is given at the bottom left corner of each map.
(0.29 MB TIF)

Figure S2 **The quality of place cell spatial firing is similar in the stationary and rotating conditions.** (A) Firing rate maps of 13 cells recorded together during a two-frame avoidance session that was flanked by two sessions of place avoidance on the stationary arena. During rotation the spatial firing of these cells was better organized in the arena frame than in the room frame. (B) The proportion of place cells with spatial coherence greater than 0.4 is similar in the stationary and rotating conditions. The 0.4 threshold was chosen because cells with spatial coherence greater than 0.4 are typically considered high quality place cells.
(1.47 MB TIF)

Figure S3 **Spatial activity of a cell with firing that is modulated by both spatial frames.** (A) Activity during the whole session is represented in the leftmost column. The other six columns represent activity during different 60° ranges (represented by an arc) of the arena displacement during rotation. This cell fired in the southwest part of the room and in the southeast part of the arena (highlighted by the green ellipse). (B) The spatial frame preference was quantified by momentary positional information (*I_pos_*). The time series of *I_pos_* from a single cell in the room frame (red) and *I_pos_* in the arena frame (blue) are shown together with the firing rate (black). The firing rate maps of the cell are shown to the right for both the room and arena frames. The spatial coherence of the rate maps is indicated, as well as the average difference of the *I_pos_* in the two frames.
(0.62 MB TIF)

Figure S4 **The relationship between ongoing ensemble** ***I_pos_*** **and the accuracy of reconstructing the rat's position from current ensemble activity.** (A) Predicting the rat's position was more accurate at the moments when ensemble *I_pos_* was large. This was true for both spatial frames. The left plot shows data from reconstructing positions in the room frame, the middle plot for the arena frame, and the rightmost plot for either of the two frames. (B) The accuracy of predicting the rat's position was greater at the moments of high ensemble *I_pos_* in every session with an ensemble of 10 or more cells. The median prediction error is shown for each session for periods of low and high ensemble *I_pos_*. The accuracy of prediction was similar to previous reports [7].
(0.52 MB TIF)

Figure S5 **The ensemble preference for one spatial frame dominates two-frame avoidance sessions.** (A) Simultaneously recorded cell pairs were more likely to have a concordant frame preference than a discordant frame preference. This result is unexpected if a cell's frame preference is independent of the preference of other cells. Spatial coherence was used to categorize a cell's frame preference, and the tendency for an excess of concordant cell pairs was observed when all cell pairs (*z* = 3.71, *p*<0.0005), only cell pairs from the same tetrode (*z* = 2.04, *p*<0.05), or only cell pairs from different tetrodes (*z* = 3.31, *p*<0.005) were analyzed. (B) A similar tendency was observed when *I_pos_* was used to assess the frame preference. Only cells with a significant preference for one of the frames were included in this analysis. The tendency for an excess of concordant cell pairs was observed when all cell pairs (*z* = 4.13, *p*<0.0005), only cell pairs from the same tetrode (*z* = 3.30, *p*<0.005), or only cell pairs from different tetrodes (*z* = 2.5, *p*<0.05) were analyzed.
(0.21 MB TIF)

Figure S6 **An ensemble's frame preference can change across environments.** Three ensembles were recorded in two different environments. (A1) Plotting the number of cells with a preference for the ensemble's preferred and non-preferred frames reveals that within each recording, the majority of cells preferentially responded to locations in the same spatial frame. (A2) Plotting the same data according to room-frame and arena-frame preference reveals that the preferred ensemble frame in the two environments could be the same (Ensemble 2) or different (Ensembles 1 and 3). (B) Ensemble cell pairs had an excess tendency to express concordant frame preferences within the same environment (*z* = 2.84; **p<0.01), but not between different environments (*z* = 1.02; *p* = 0.31; same versus different environment χ^2^ = 7.42; *p*<0.01).
(0.36 MB TIF)

Figure S7 **The waveform discrimination of the units shown in** **Figure 3A** **.** Cells recorded from two tetrodes were discriminated based on the amplitude and other parameters of the waveform on the four tetrode channels. Example action potential waveforms are shown on the right.
(1.25 MB TIF)

Figure S8 **The waveform discrimination of the units shown in** **Figure 3B** **.** Cells recorded from two tetrodes were discriminated based on the amplitude and other parameters of the waveform on the tetrode channels. Example action potential waveforms are shown on the right.
(1.06 MB TIF)

Figure S9 **Ensemble dynamics of frame preference during two-frame avoidance, as shown on** **Figure 2F** **, zoomed in on a shorter timescale.** Ensemble *I_pos_* was computed as the difference between room and arena *I_pos_* during 117 ms intervals for each cell summed across all cells in a single recording. Positive values (red) indicate ensemble preference for the room frame; negative values (blue) indicate preference for the arena frame. Figures A, B, and C show data from three different recordings. Panels A and B are from the same recordings as Figure 2F1 and 2F2, respectively.
(0.34 MB TIF)

Figure S10 **Comparison of session-averaged spatial frame preferences and session-averaged behavior.** (Left) The proportion of room and arena preferring cells during two-frame avoidance sessions was not related to the relative proportion of entrances to the room shock zone and the arena shock zone. (Right) The average relative distance to the two shock zones was also not related to the ensemble spatial frame preference.
(0.24 MB TIF)

Figure S11 **Three parameters that were used to quantify spatially selective firing of place cells.** (A) Firing rate maps of seven neurons, these neurons were chosen so that cells with well-organized firing fields and cells with spatially disorganized firing are represented. The scatter plots in (B) and (C) show spatial coherence, mean momentary positional information (*I_pos_*), and information content of all recorded cells. The points representing the neurons shown in (A) are marked by colors corresponding to colors of the squares around the firing rate maps. These data suggest that the coherence and *I_pos_* values better reflect the intuitive evaluation of firing field quality than values of information content. Panels D–E show the relationship between the spatial firing measures and the firing rate of a cell. The figure shows that spatial coherence (D) and mean *I_pos_* (F) are positively correlated with the firing rate, while information content (E) is strongly negatively correlated with firing rate.
(0.72 MB TIF)

Table S1 10.1371/journal.pbio.1000403Average and SEM values are given. *Spatial similarity was only analyzed in units that had spatially well-organized firing in at least one condition (coherence>0.4 was used as a criterion for well-organized firing). **Only neurons with a firing field were analyzed.
(0.04 MB DOC)

Text S1 **Detailed descriptions and discussions are provided on the following topics.** The temporal organization of cell discharge according to spatial frame; double rotation experiments; anatomical grouping in hippocampus; the single cell firing characteristics; the characteristics of room-preferring and arena-preferring ensemble states; fear during the two-frame task; momentary positional information (*I_pos_*); and computing *I_pos_*.
(0.18 MB DOC)

Video S1 **Illustration of the rat's behavior in the two-frame task.** The arena and the rat are viewed from above. The room and arena shock zones are visualized as red and blue areas for the purpose of presentation, but these color areas were not available for the rat. The rat had to use arena and room landmarks for its avoidance behavior.
(4.78 MB MOV)

## Abbreviations

LED: light emitting diode
TTX: tetrodotoxin
