# Correction: Dynamic Grouping of Hippocampal Neural Activity During Cognitive Control of Two Spatial Frames

**DOI:** 10.1371/journal.pbio.1002100

**Published:** 2015-03-11

**Authors:** The PLOS Biology Staff

In S2 Fig., the second and third rows of firing rate maps in panel A are swapped and incorrectly labeled "arena frame" and "room frame" respectively. Instead, the second row should be labeled “room frame” and the third row should be labeled “arena frame”. Also, the spatial coherence numbers for the fourth row of firing rate maps in panel A were incorrectly duplicated from the first row. Please see the corrected S2 Fig. here.

The figure legend also omits the sentence “Spatial coherence is given below each firing rate map.” Please view the corrected figure and legend below.

## Supporting Information

S2 Fig The quality of place cell spatial firing is similar in the stationary and rotating conditions. (A) Firing rate maps of 13 cells recorded together during a two-frame avoidance session that was flanked by two sessions of place avoidance on the stationary arena. During rotation the spatial firing of these cells was better organized in the arena frame than in the room frame. Spatial coherence is given below each firing rate map. (B) The proportion of place cells with spatial coherence greater than 0.4 is similar in the stationary and rotating conditions. The 0.4 threshold was chosen because cells with spatial coherence greater than 0.4 are typically considered high quality place cells.
(TIF)

## References

[1] Kelemen E, Fenton AA (2010) Dynamic Grouping of Hippocampal Neural Activity During Cognitive Control of Two Spatial Frames. PLoS Biol 8(6): e1000403 doi: 10.1371/journal.pbio.1000403 20585373 10.1371/journal.pbio.1000403 PMC2889929

