# Supplementary figures and images for: Dynamic Grouping of Hippocampal Neural Activity During Cognitive Control of Two Spatial Frames
Source: PLoS Biol. 2010 Jun 22;8(6):e1000403. doi: 10.1371/journal.pbio.1000403 (PMC2889929; doi:10.1371/journal.pbio.1000403)

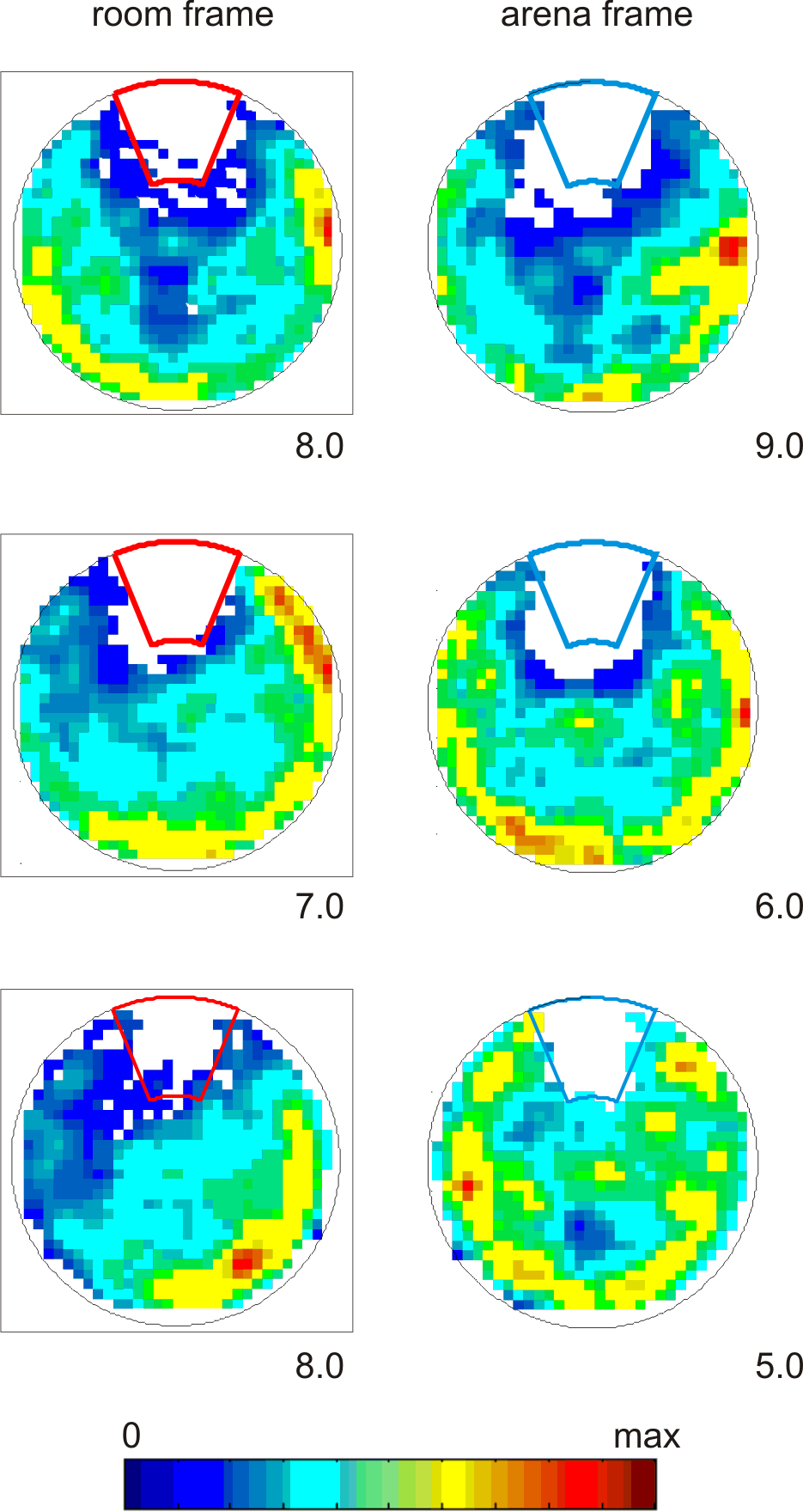

Supplement: Figure S1 — Time spent in different parts of the arena and room during three different sessions of the two-frame task. The time spent in each pixel is color coded. The dwell time in seconds of the most visited pixel is given at the bottom left corner of each map. (0.29 MB TIF) [file pbio.1000403.s001.tif]

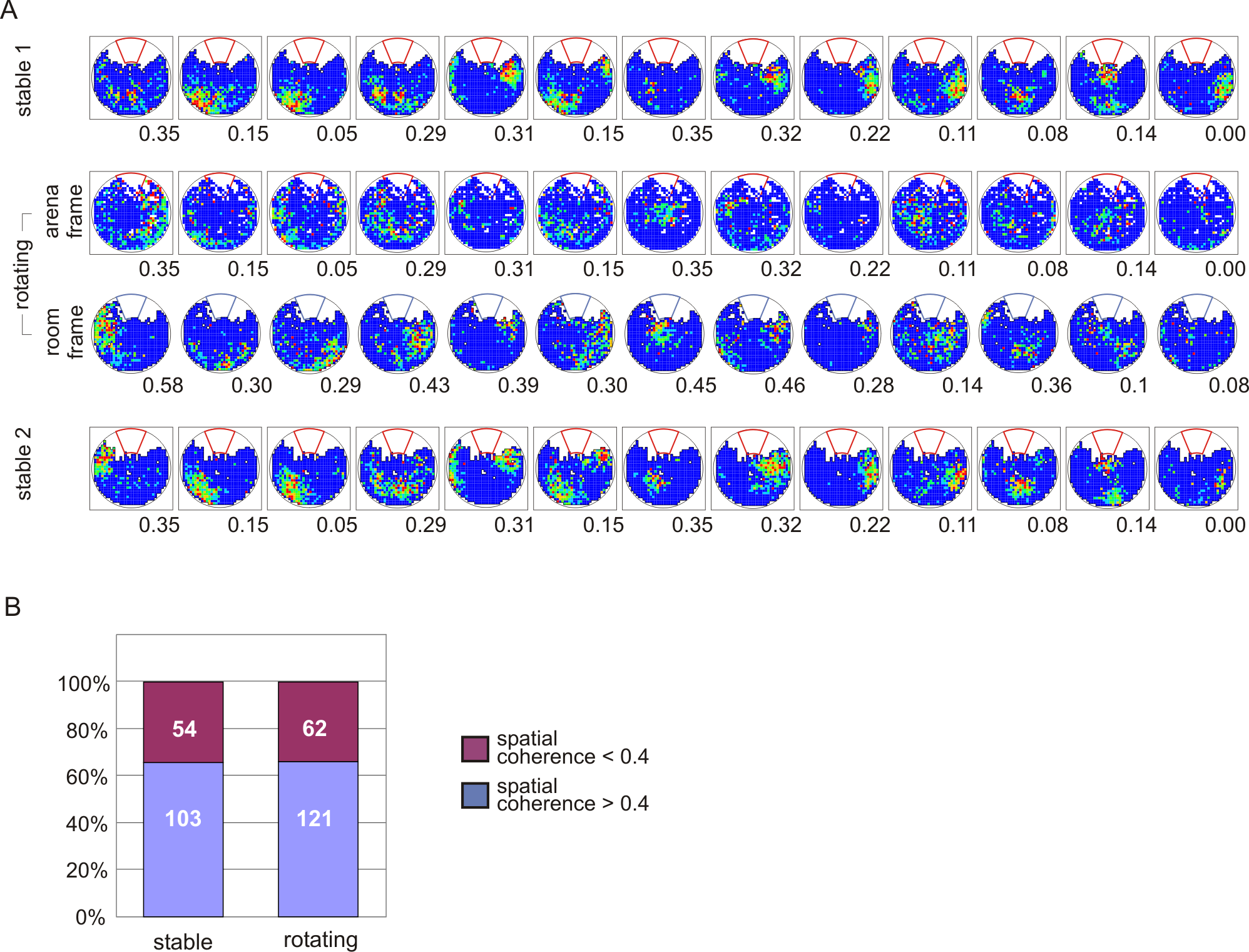

Supplement: Figure S2 — The quality of place cell spatial firing is similar in the stationary and rotating conditions. (A) Firing rate maps of 13 cells recorded together during a two-frame avoidance session that was flanked by two sessions of place avoidance on the stationary arena. During rotation the spatial firing of these cells was better organized in the arena frame than in the room frame. (B) The proportion of place cells with spatial coherence greater than 0.4 is similar in the stationary and rotating conditions. The 0.4 threshold was chosen because cells with spatial coherence greater than 0.4 are typically considered high quality place cells. (1.47 MB TIF) [file pbio.1000403.s002.tif]

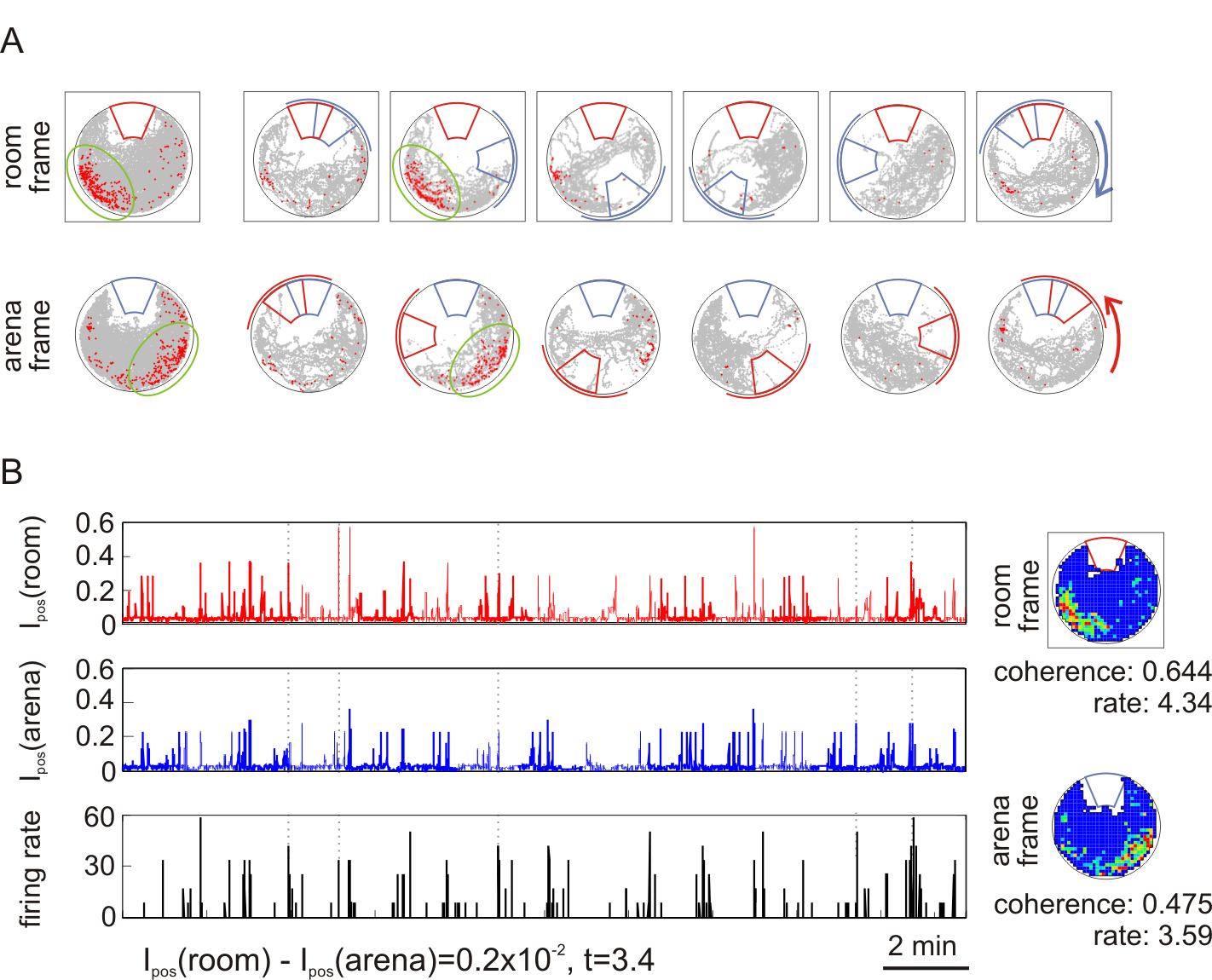

Supplement: Figure S3 — Spatial activity of a cell with firing that is modulated by both spatial frames. (A) Activity during the whole session is represented in the leftmost column. The other six columns represent activity during different 60° ranges (represented by an arc) of the arena displacement during rotation. This cell fired in the southwest part of the room and in the southeast part of the arena (highlighted by the green ellipse). (B) The spatial frame preference was quantified by momentary positional information (Ipos). The time series of Ipos from a single cell in the room frame (red) and Ipos in the arena frame (blue) are shown together with the firing rate (black). The firing rate maps of the cell are shown to the right for both the room and arena frames. The spatial coherence of the rate maps is indicated, as well as the average difference of the Ipos in the two frames. (0.62 MB TIF) [file pbio.1000403.s003.tif]

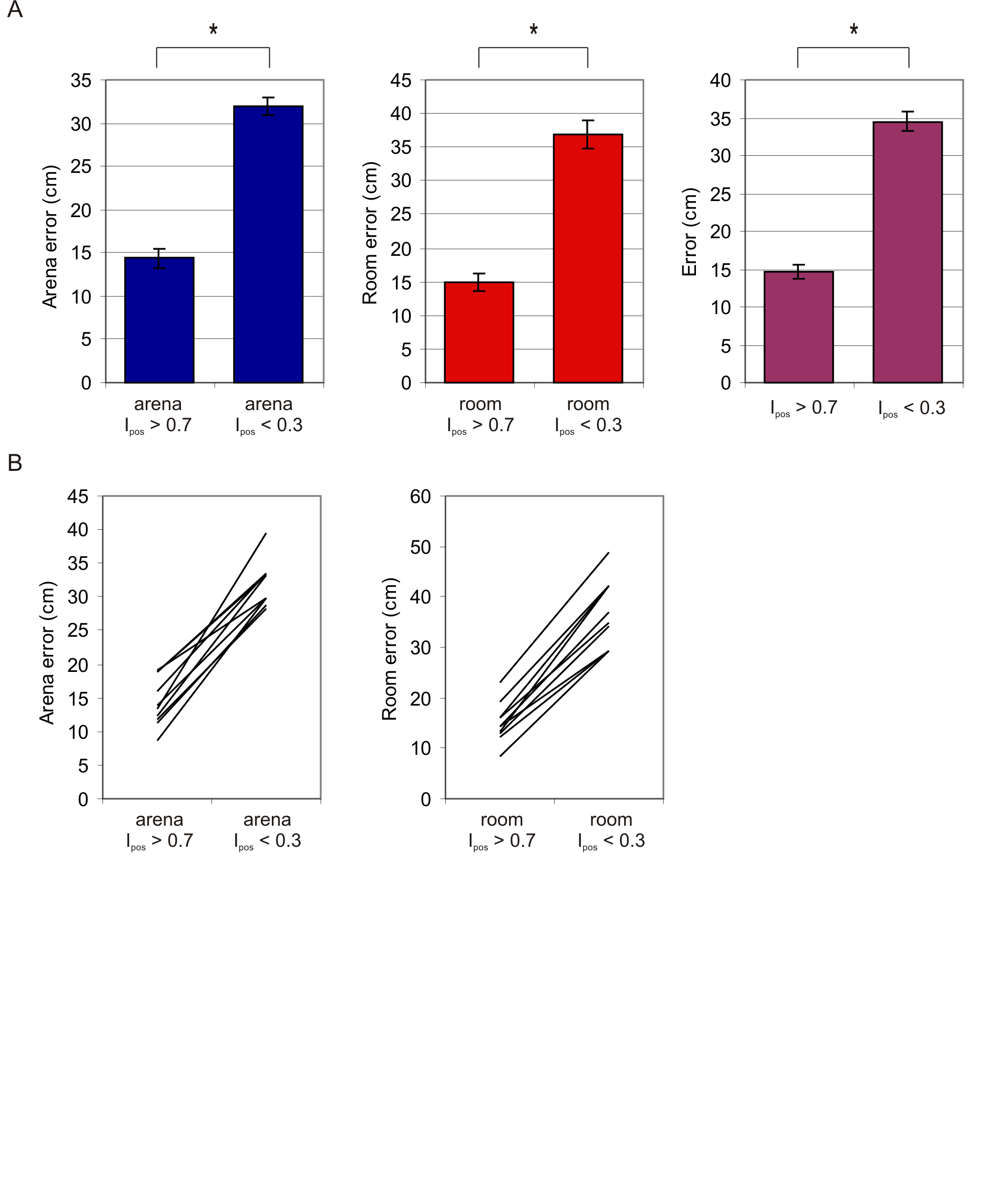

Supplement: Figure S4 — The relationship between ongoing ensemble Ipos and the accuracy of reconstructing the rat's position from current ensemble activity. (A) Predicting the rat's position was more accurate at the moments when ensemble Ipos was large. This was true for both spatial frames. The left plot shows data from reconstructing positions in the room frame, the middle plot for the arena frame, and the rightmost plot for either of the two frames. (B) The accuracy of predicting the rat's position was greater at the moments of high ensemble Ipos in every session with an ensemble of 10 or more cells. The median prediction error is shown for each session for periods of low and high ensemble Ipos. The accuracy of prediction was similar to previous reports [7]. (0.52 MB TIF) [file pbio.1000403.s004.tif]

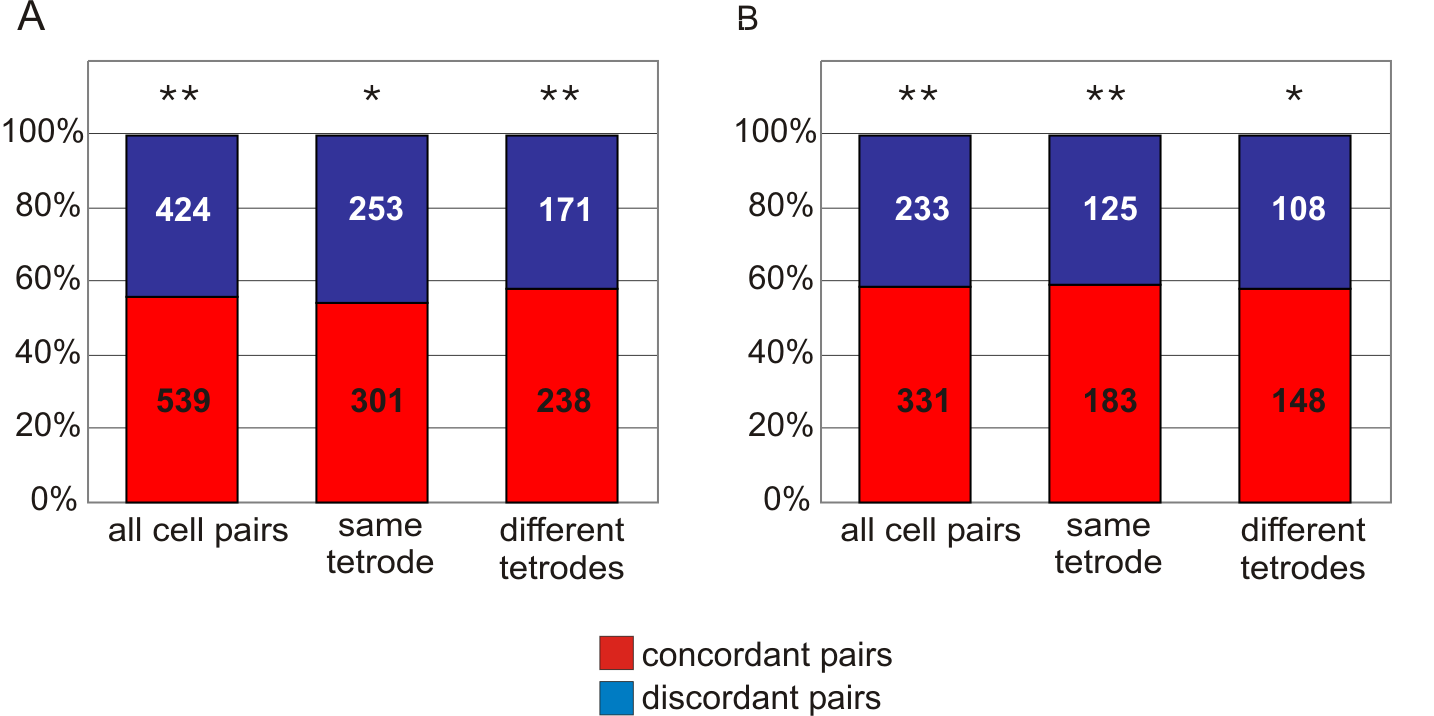

Supplement: Figure S5 — The ensemble preference for one spatial frame dominates two-frame avoidance sessions. (A) Simultaneously recorded cell pairs were more likely to have a concordant frame preference than a discordant frame preference. This result is unexpected if a cell's frame preference is independent of the preference of other cells. Spatial coherence was used to categorize a cell's frame preference, and the tendency for an excess of concordant cell pairs was observed when all cell pairs (z = 3.71, p<0.0005), only cell pairs from the same tetrode (z = 2.04, p<0.05), or only cell pairs from different tetrodes (z = 3.31, p<0.005) were analyzed. (B) A similar tendency was observed when Ipos was used to assess the frame preference. Only cells with a significant preference for one of the frames were included in this analysis. The tendency for an excess of concordant cell pairs was observed when all cell pairs (z = 4.13, p<0.0005), only cell pairs from the same tetrode (z = 3.30, p<0.005), or only cell pairs from different tetrodes (z = 2.5, p<0.05) were analyzed. (0.21 MB TIF) [file pbio.1000403.s005.tif]

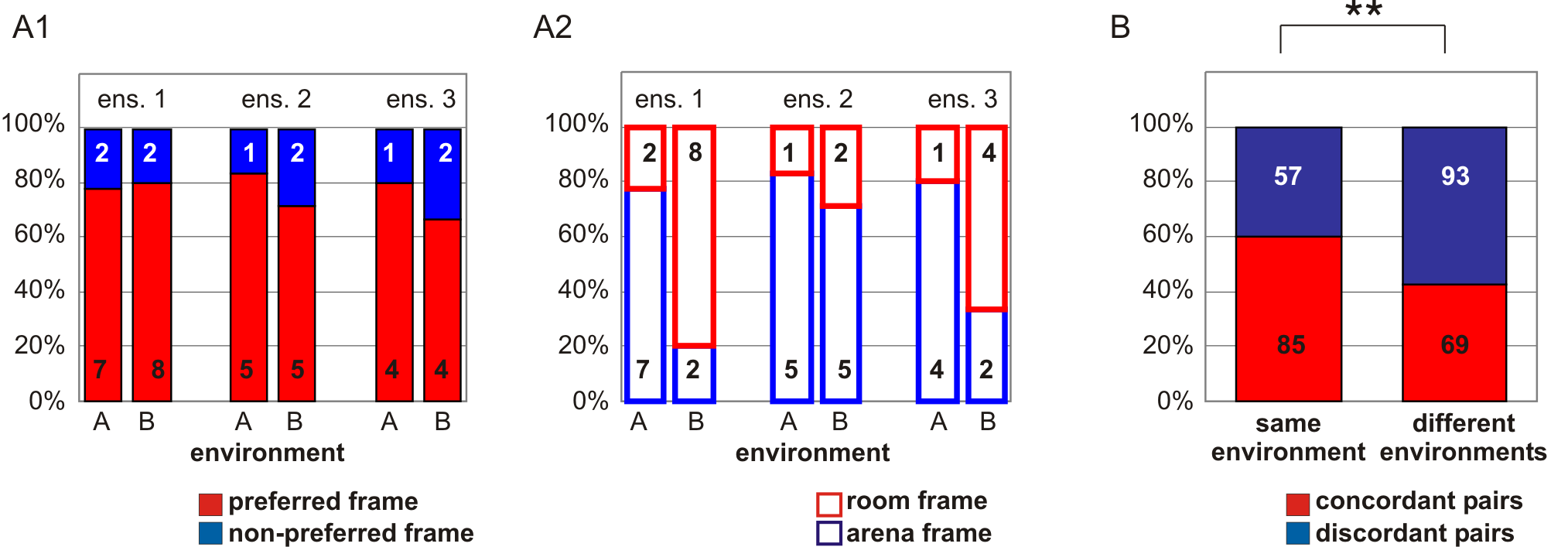

Supplement: Figure S6 — An ensemble's frame preference can change across environments. Three ensembles were recorded in two different environments. (A1) Plotting the number of cells with a preference for the ensemble's preferred and non-preferred frames reveals that within each recording, the majority of cells preferentially responded to locations in the same spatial frame. (A2) Plotting the same data according to room-frame and arena-frame preference reveals that the preferred ensemble frame in the two environments could be the same (Ensemble 2) or different (Ensembles 1 and 3). (B) Ensemble cell pairs had an excess tendency to express concordant frame preferences within the same environment (z = 2.84; **p<0.01), but not between different environments (z = 1.02; p = 0.31; same versus different environment χ2 = 7.42; p<0.01). (0.36 MB TIF) [file pbio.1000403.s006.tif]

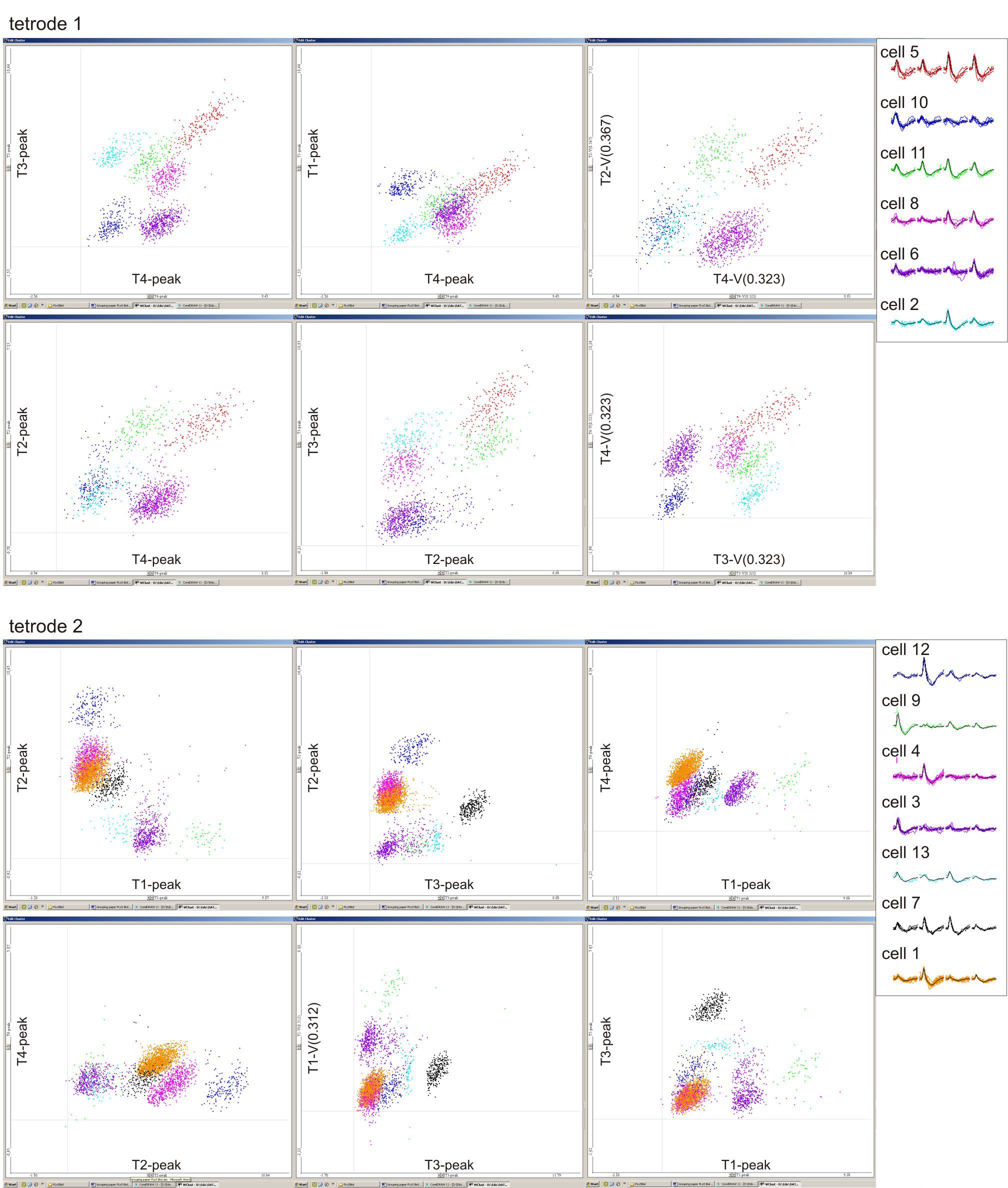

Supplement: Figure S7 — The waveform discrimination of the units shown in Figure 3A . Cells recorded from two tetrodes were discriminated based on the amplitude and other parameters of the waveform on the four tetrode channels. Example action potential waveforms are shown on the right. (1.25 MB TIF) [file pbio.1000403.s007.tif]

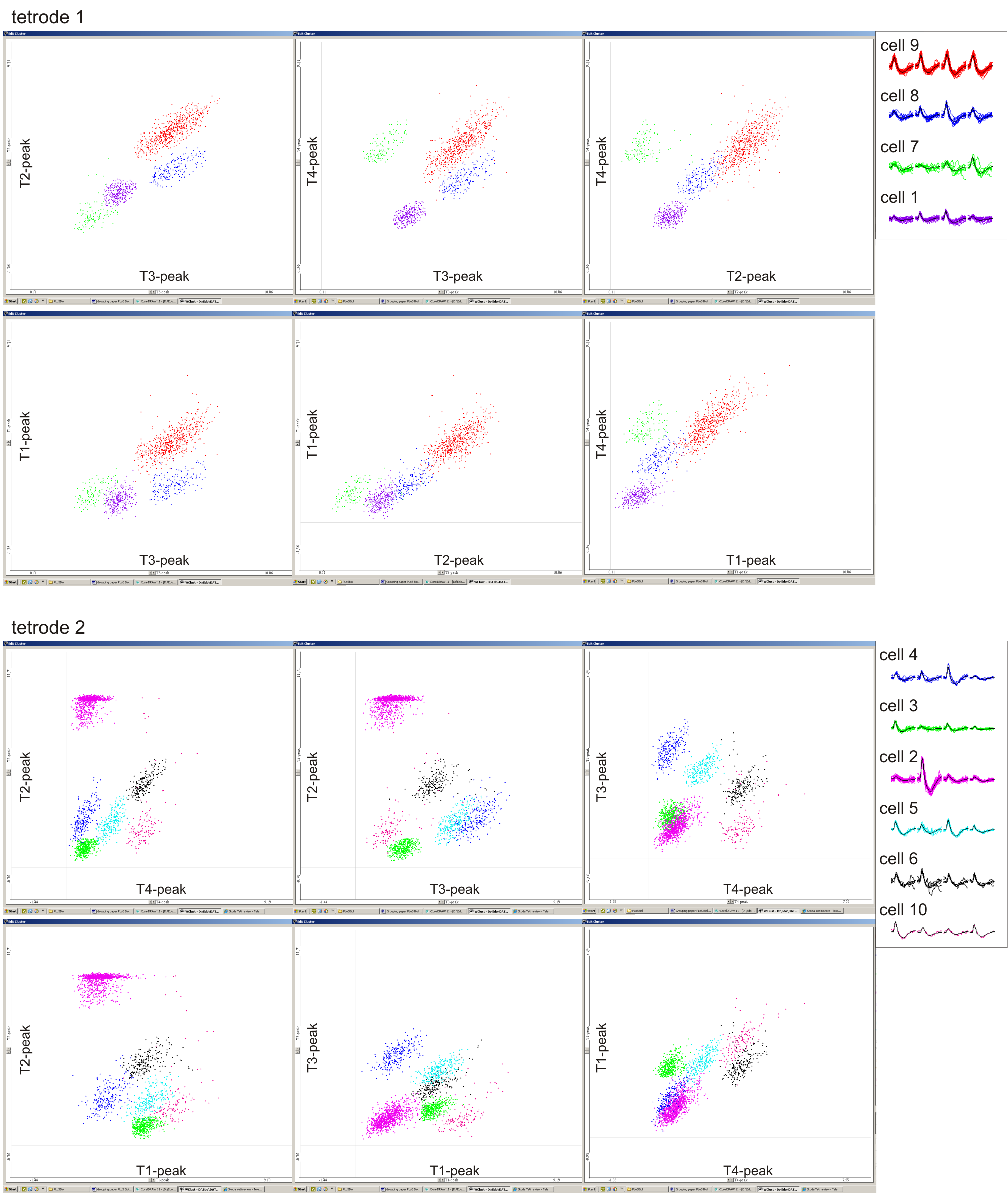

Supplement: Figure S8 — The waveform discrimination of the units shown in Figure 3B . Cells recorded from two tetrodes were discriminated based on the amplitude and other parameters of the waveform on the tetrode channels. Example action potential waveforms are shown on the right. (1.06 MB TIF) [file pbio.1000403.s008.tif]

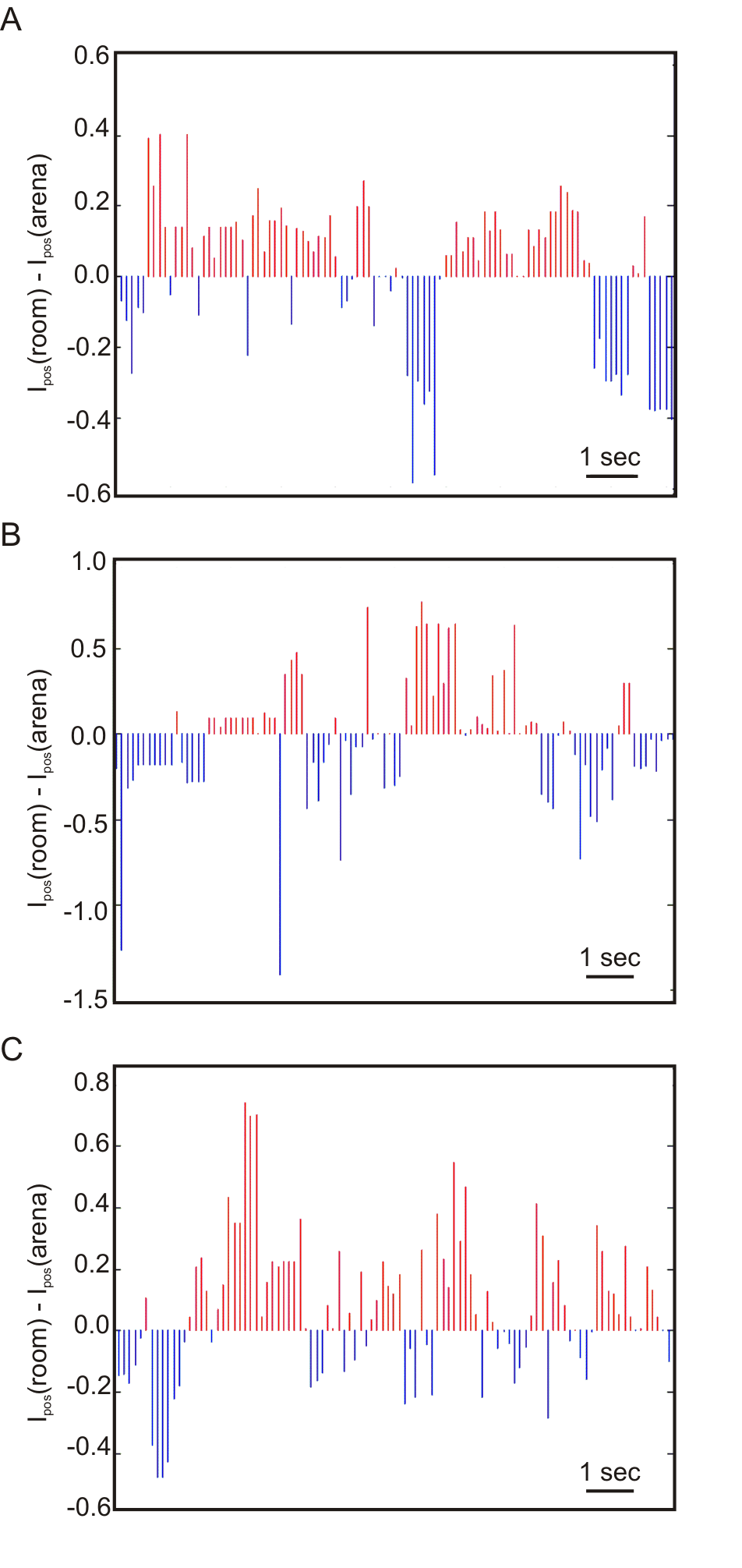

Supplement: Figure S9 — Ensemble dynamics of frame preference during two-frame avoidance, as shown on Figure 2F , zoomed in on a shorter timescale. Ensemble Ipos was computed as the difference between room and arena Ipos during 117 ms intervals for each cell summed across all cells in a single recording. Positive values (red) indicate ensemble preference for the room frame; negative values (blue) indicate preference for the arena frame. Figures A, B, and C show data from three different recordings. Panels A and B are from the same recordings as Figure 2F1 and 2F2, respectively. (0.34 MB TIF) [file pbio.1000403.s009.tif]

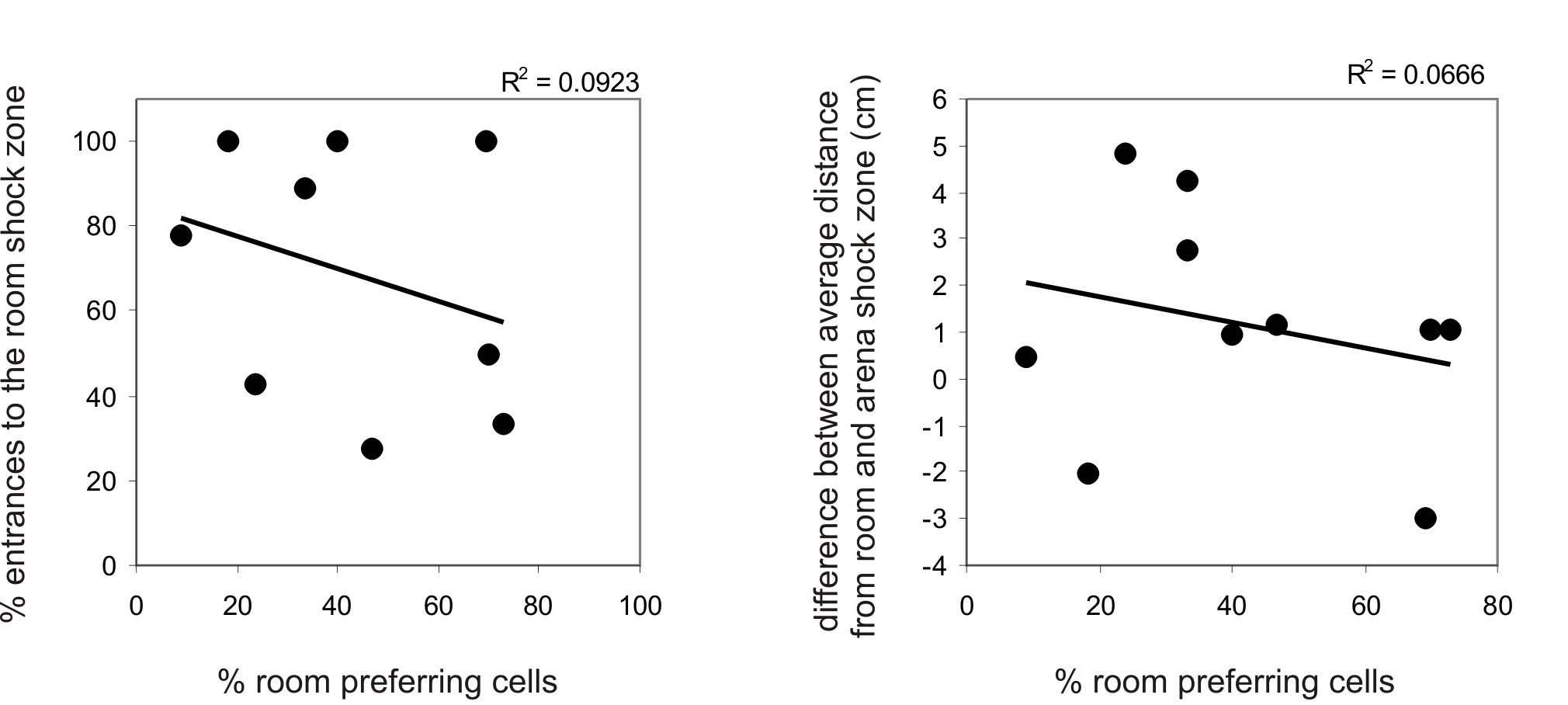

Supplement: Figure S10 — Comparison of session-averaged spatial frame preferences and session-averaged behavior. (Left) The proportion of room and arena preferring cells during two-frame avoidance sessions was not related to the relative proportion of entrances to the room shock zone and the arena shock zone. (Right) The average relative distance to the two shock zones was also not related to the ensemble spatial frame preference. (0.24 MB TIF) [file pbio.1000403.s010.tif]

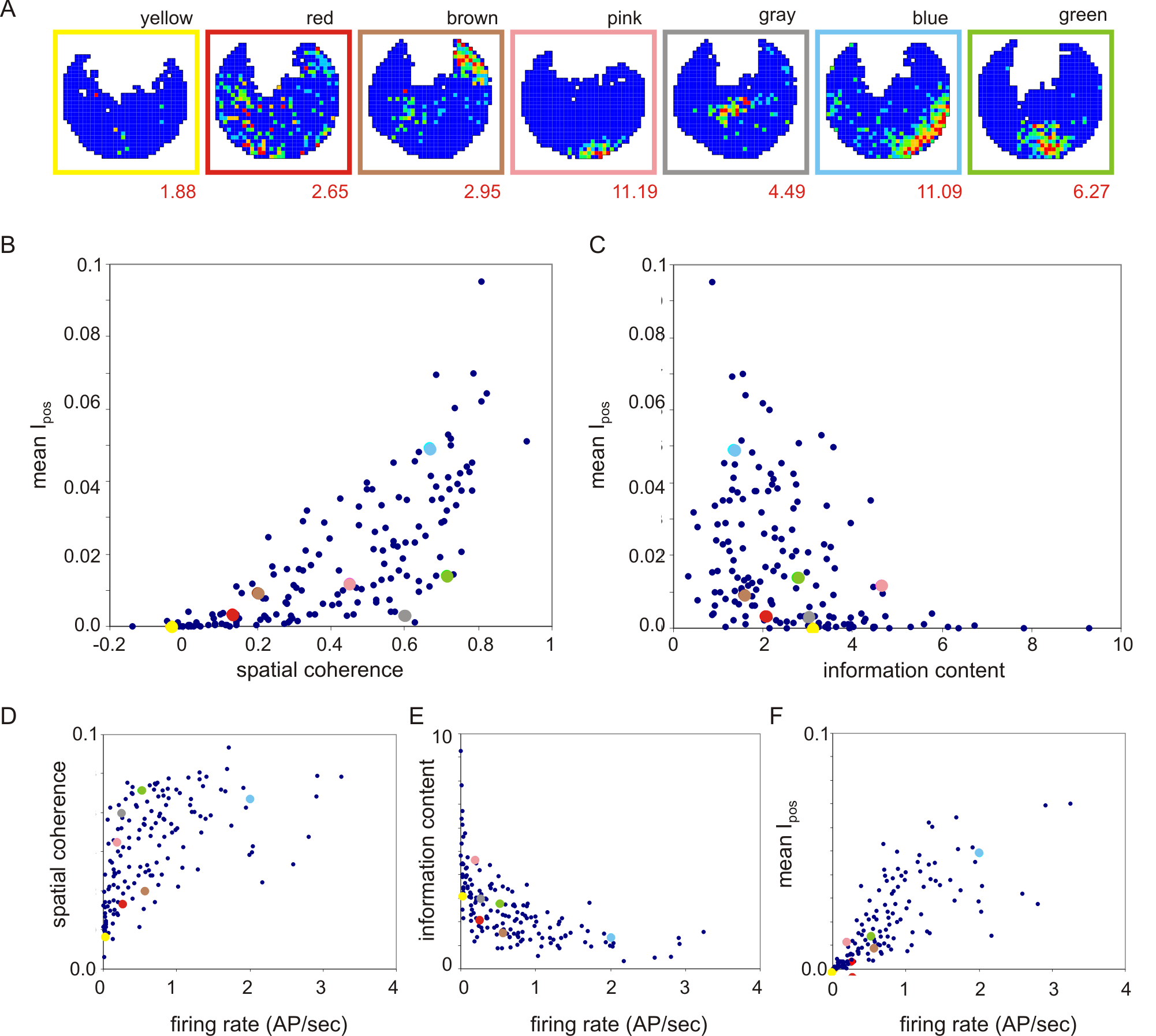

Supplement: Figure S11 — Three parameters that were used to quantify spatially selective firing of place cells. (A) Firing rate maps of seven neurons, these neurons were chosen so that cells with well-organized firing fields and cells with spatially disorganized firing are represented. The scatter plots in (B) and (C) show spatial coherence, mean momentary positional information (Ipos), and information content of all recorded cells. The points representing the neurons shown in (A) are marked by colors corresponding to colors of the squares around the firing rate maps. These data suggest that the coherence and Ipos values better reflect the intuitive evaluation of firing field quality than values of information content. Panels D–E show the relationship between the spatial firing measures and the firing rate of a cell. The figure shows that spatial coherence (D) and mean Ipos (F) are positively correlated with the firing rate, while information content (E) is strongly negatively correlated with firing rate. (0.72 MB TIF) [file pbio.1000403.s011.tif]
