# Supplementary material for: Dynamic Grouping of Hippocampal Neural Activity During Cognitive Control of Two Spatial Frames
Source: PLoS Biol. 2010 Jun 22;8(6):e1000403. doi: 10.1371/journal.pbio.1000403 (PMC2889929; doi:10.1371/journal.pbio.1000403)
Supplement: Table S1 — 10.1371/journal.pbio.1000403Average and SEM values are given. *Spatial similarity was only analyzed in units that had spatially well-organized firing in at least one condition (coherence>0.4 was used as a criterion for well-organized firing). **Only neurons with a firing field were analyzed. (0.04 MB DOC) [file pbio.1000403.s012.doc]

| Property | Stable | Rotating: preferred frame  (comparison vs. stable) | Rotating: non-preferred frame  (comparison vs. stable)  (paired comparison vs. preferred frame) |
| --- | --- | --- | --- |
| Number of active pyramidal neurons recorded | 157 | 183  χ^2^=9.50, p<0.05 | 183  χ^2^=9.50, p<0.05 |
| Overall rate (AP/s) | 0.86 + 0.06 | 0.79 + 0.06  t_338_=0.78, p=0.44 | 0.79 + 0.06  t_338_=0.78, p=0.44 |
| Spatial similarity to stable*  (std units) | 0.39 + 0.026 | 0.16 + 0.014  t_213_=8.73, p<0.001 | 0.03 + 0.010  t_213_=15.43, p<0.001  t_135_=11.42, p<0.001 |
| average *I_pos_*  (bits) | 0.022 + 0.0015 | 0.013 + 0.0009  t_338_=5.42, p<0.0001 | 0.009 + 0.0007  t_338_=8.33, p<0.0001  t_182_=10.19, p<0.0001 |
| Coherence  (std units) | 0.48 + 0.02 | 0.46 + 0.01  t_338_=0.90, p=0.37 | 0.32 + 0.01  t_338_=7.35, p<0.0001  t_182_=15.74, p<0.01 |
| Center rate**  (AP/s) | 9.68 + 0.52 | 8.86 + 0.61  t_333_=1.00, p=0.32 | 5.67 + 0.39  t_331_=6.28, p<0.0001  t_180_=9.62, p<0.0001 |
| Information content (bits/AP) | 2.13 + 0.08 | 1.78 + 0.06  t_338_=3.57, p=0.0004 | 1.53 + 0.05  t_338_=6.60, p<0.0001  t_182_=11.58, p<0.0001 |
| Active proportion of arena | 0.24 + 0.01 | 0.31 + 0.01  t_338_=-4.71, p<0.0001 | 0.34 + 0.01  t_338_=-6.40, p<0.0001  t_182_=14.11, p<0.01 |

Table S1. Discharge properties of recorded complex-spike cells. Average and SEM values are given. *Spatial similarity was only analyzed in units that had spatially well-organized firing in at least one condition (coherence>0.4 was used as a criterion for well-organized firing.). **Only neurons with a firing field were analyzed.
