# Supplementary material for: Dynamic Grouping of Hippocampal Neural Activity During Cognitive Control of Two Spatial Frames
Source: PLoS Biol. 2010 Jun 22;8(6):e1000403. doi: 10.1371/journal.pbio.1000403 (PMC2889929; doi:10.1371/journal.pbio.1000403)
Supplement: Text S1 — Detailed descriptions and discussions are provided on the following topics. The temporal organization of cell discharge according to spatial frame; double rotation experiments; anatomical grouping in hippocampus; the single cell firing characteristics; the characteristics of room-preferring and arena-preferring ensemble states; fear during the two-frame task; momentary positional information (Ipos); and computing Ipos. (0.18 MB DOC) [file pbio.1000403.s013.doc]

**Dynamic grouping of hippocampal neural activity during cognitive control of two spatial frames**

**Text S1**

*Temporal organization of cell discharge according to spatial frame*

The idea that cells participating in the same representation should discharge together in time was suggested as a solution to the binding problem, and a way to avoid the superposition catastrophe [1,2,3,4]. Consistent with this hypothesis, we observed a dynamic, functional grouping of active CA1 neurons according to spatial frame during the two-frame avoidance task. The tendency for cells with the same frame preference to discharge together was most prominent at timescales shorter than a second, similar to the timescale of switching spatial representations during foraging in stationary environments [5,6].

The organization of CA1 discharge on the sub-second timescales of theta and gamma oscillations was not unexpected. Theta organizes hippocampus discharge such that the timing of spikes relative to the phase of theta may provide information about the position of the rat in an environment [7,8] and helps to organize the activity of neurons into temporal sequences [8]. Coalitions of hippocampus cells firing together on the timescale of 20-30 ms were identified and called “cell assemblies” [9]. Our data suggest that when two representations are concurrently relevant, dynamic functional grouping, a temporal coordination mechanism, organizes the discharge of cells that represent the same spatial frame to discharge together in time and separate in time from discharge that represents information within a different spatial frame. We also showed that in open field environments the similarity of spatial discharge is also related to temporal coordination – the more similar the firing fields of a pair of cells, the closer in time the neurons tend to discharge, a phenomenon called “sequence compression” that has been characterized in linear track environments [8,10]. These data suggest that the temporal organization of CA1 discharge at sub-second timescales may serve two distinct purposes by coordinating discharge on two levels of spatial organization. At the first level, as has been shown previously for linear track environments, discharge signalling locations within the same spatial frame is organized according to the spatial locations the cells encode. This temporal organization may provide a means to link sequences of locations into encoded and recalled trajectories as an animal traverses space [11]. At the second level, the present work shows that CA1 discharge is organized at multiple time scales according to distinct spatial frames. This temporal organization may serve to segregate the processing of distinct categories of information within a single network, thereby reducing the chances for interference between separate information streams.

Attractor network models and cell assembly hypotheses dominate thinking about the organization of hippocampus network discharge [9,12,13,14]. Our observation that subsets of neurons do not discharge independently is consistent with these models, and ensemble coding hypotheses in general [15]. In addition to being a correlate of cognitive control, the continuous switching of the ensemble frame preference in accord with purposeful two-frame behaviour is also in accord with cell assembly hypotheses of hippocampus operation. Such switching between spatial representations would not however be consistent with an attractor model in its purest form, where the same attractor corresponds to a single spatial map that is active during an entire recording session [12]. The switching we observed is however consistent with ‘maplet’ models in which multiple attractor states can be observed under steady-state behavioural conditions [14]. Independent of the specifics of any formal model, the dynamic functional grouping we observed is consistent with the general notion that hippocampus activity operates as a cohesive network that can rapidly switch between different attractor-like states to represent different categories of information during a single session.

Injecting TTX into one dorsal hippocampus selectively disrupts the timing of action potentials between different CA1 cells in the uninjected hippocampus [16] and as shown in Fig. 1 of the main text, the same manipulation also impairs the ability to do and learn tasks when two distinct spatial frames must be coordinated [17,18,19]. The present finding of a dynamic functional grouping of hippocampal discharge is predicted by these previous findings. Together this work combining functional lesions, cognitive behavior, and in vivo electrophysiology extends the established notion that hippocampus is important for storing information in memory, by providing compelling evidence that the timing of action potentials between different CA1 cells is crucial for the coordinated processing of distinct representations of information, in addition to the role of hippocampus in memory storage [20,21,22].

### Double rotation experiments

A series of experiments from different laboratories were undertaken to investigate whether the discharge within an ensemble of CA1 neurons is independent as predicted by dedicated, local-coding hypotheses or whether the discharge is cohesive as predicted by distributed, ensemble-coding and attractor-network hypotheses [23,24,25,26,27]. These so-called “double rotation experiments” had a similar design. On rare probe sessions, two sets of landmarks were rotated in opposite directions to create a cue conflict condition relative to the baseline recording sessions with the cues in standard locations. The responses of place cells to this conflict manipulation were recorded and compared to baseline firing. The experiments did not lead to conclusive results; both independent and cohesive responses were reported in CA1.

The approach we took in the present investigation followed from the double rotation studies, adding three major improvements. We studied CA1 responses in steady-state cue-conflict conditions, rather than on probe trials. In prior double rotation experiments cell responses changed between the two sessions in a way similar to remapping [24,26], making the results difficult to interpret. Using the two-frame task allowed us to study place cells in steady state cue conflict conditions, within a single recording session, so between-session comparisons to baseline were unnecessary.

A second way in which our study differed from prior double rotation experiments is that prior work averaged the individual place cell responses. Averaging the activity of individual cells across an entire session precluded analysis of the coordinated ensemble responses as well as the dynamic changes in hippocampal discharge within a session. Accumulating evidence suggests that hippocampal discharge changes in time, even within recording sessions in stationary environmental conditions [5,6,28,29]. Such dynamic changes of activity would not have been noticed because the activity was averaged in prior work. In our analysis we concentrated on the dynamics of hippocampal discharge on timescales as short as tens of milliseconds and the interactions between cells. The results we obtained support the idea that different spatial frames are alternatively represented in cohesive, multi-stable CA1 ensemble activity patterns that emerge at different times with the one of the frames dominating a given session.

Our experiment differed from the double rotation experiments in a third potentially important way. In our paradigm the rats were reinforced to organize their behavior in the two spatial frames, while in prior work there was no explicit reinforcement for individually processing the two sets of landmarks. This allowed us to relate the changes between multi-stable ensemble representations of spatial frames to the rat’s ongoing, purposeful behavior.

Prior work studied the activity of cells relative to different spatial frames [30,31,32]. In addition to place cells which fired in different locations of the room, cells were also observed to fire when the animal was close to a goal location or to the start and finish box, regardless of the position in the environment. The results were very similar to the observation that place cells that discharge near to a movable barrier tends to move together with the barrier when it is repositioned [33,34]. Unfortunately, in these studies the data were averaged over the entire session, making it impossible to trace short timescale changes in network dynamics.

In contrast to the double rotation studies, in our experiments there were two spatial frames, both of which were defined by distributed sets of landmarks that were present throughout the experiment with effectively similar salience. However, it was not the salience, but the momentary behavioral relevance of one or the other spatial frame that organized the dynamic changes in which of the two spatial frames would be represented at each instant. There was a strong tendency for groups of coactive cells to signal the same type of information. We also observed a tendency of the entire network to switch between the two spatial frames to preferentially process information according to the momentary behavioral needs. These recordings provided direct evidence for a role of hippocampus in coordinating two streams of information. The exact nature of the hippocampal role in cognitive control must be left for further study. Other brain structures have been implicated and likely participate in these computations, sharing information with each other, and their exact place in the cognitive control system is still unknown.

*Anatomical grouping in hippocampus?*

A possible anatomical grouping of hippocampal cells according to their functional properties has been a matter of controversy and spirited discussions [35]. In rat hippocampus, the firing fields of place cells within ~1 mm were reportedly closer than expected by chance [36]. In a delayed-non-match-to-sample task, cells within 600 – 800 µm had similar responses. Cells were anatomically grouped according to their responses to the left or right lever-presses, or according to the phase of the task [35]. Although a thorough study of over 3000 place cells failed to reveal any similarity between firing fields of anatomically adjacent cells on a linear track [37], an in situ hybridization study of immediate early gene products provided evidence that co-active cells may form small clusters of about 4 cells in the hippocampus [38].

Although this anatomical grouping controversy was not the topic of our study, our results may have some relevance. The vast majority of cells we recorded were not further than 600 µm from each other. We saw coherent frame preference in cells within this distance, which resembles the results suggesting anatomical grouping [39]. The cohesive responses were observed at the level of spatial frame, but not at the level of location. If functional grouping exists at the level of spatial frame, and not at the level of locations within a frame, this could partly explain the discrepancy [37,39].

In summary, our data show that within the CA1 network, just one representation is predominantly active at any particular time. The tendency of the CA1 network to represent one spatial frame at a time was observed in data averaged over the entire recording session as well as on the sub-second timescales of hippocampal theta and gamma oscillations. The dynamic functional grouping of neurons into spatial frame response categories can serve as a mechanism for coordinating distinct representations and can prevent interference between them.

*Single cell firing characteristics*

Both spatial frames were represented in CA1 discharge. For some cells, the activity was predominantly controlled by one of the frames. Figure 2A1 in the main text shows an example of a cell responding to room locations, and figure 2A2 is an example of a cell responding to the arena locations. A cell with activity modulated by locations in both spatial frames is shown in figure S3.

The co-occurrence of the two representations on a long timescale is neither surprising nor controversial given that one of main findings of the present work is that locations in both frames are represented within a session. However, the ensemble recordings also show that at any moment information about location in one or the other frame predominates (Fig. 2). Several independent methods of analysis showed that the tendency for concordant frame-specific responses increased with shorter timescales of analysis, consistent with the observation that the ensemble rapidly alternates between the spatial frames being represented at a given moment (Figs. 2E and 4).

In light of the finding of a dynamic functional grouping of hippocampal discharge during two-frame avoidance, several technical issues merit consideration. If a cell’s discharge is spatially organized in one frame, it is likely also to display some spatial organization in the other frame because discharge in the two spatial frames is not strictly independent. For example, if a cell has a firing field on the periphery of the arena, which is often the case in one frame, then the cell will fire on the periphery of the arena in the other frame too. An extreme example of this frame ambiguity are cells with firing fields in the center of the arena, where it is particularly hard to attribute activity to one or the other spatial frame. Consequently, our measures have likely overestimated the overlap between the two frame-specific representations. This was confirmed by the fact that using more strict criteria to assign the frame preference of discharge caused an apparent increased in the frame concordance of coactive cell responses (see Results in the main text).

Comparing the average *I_pos_* in each of the two frames during an entire session by the Wilcoxon test was one way to assess the frame preference of individual cells. This method revealed that the majority (75.4%) of place cells had a statistically reliable preference for location-specific discharge in one of the frames during a session of two-frame avoidance. To quantify the modulation of a cell’s firing by locations in the two frames, we also artificially created the spatial firing distribution that would be expected if the activity was merely controlled by one of the frames (let it be room frame) and computed an estimate of information, the entropy of this distribution. The observed entropy was also computed. The difference between the observed and expected entropy estimates the amount of information that the arena frame provides in addition to the room frame. In an analogous manner we computed the information provided by the room frame in addition to the arena frame. For some cells, most of the information was provided by one dominant frame and the other frame provided little extra information. But we also observed several cells in which the arena frame provided a substantial amount of information in addition to the room frame, and vice versa (Fig. S3), suggesting that the activity of these cells was modulated by both reference frames.

The time series of *I_pos_* provides a window into the time course of the modulation by the two spatial frames within a session. When the cells discharge, the ones that are predominantly modulated by only one of the frames show increased *I_pos_* only in the dominant frame (Fig 2B1 and 2B2). In contrast, the cells that had no clearly dominant frame could have increased *I_pos_* in one, or the other, or both frames at any moment. Based on these analyses nine of the 183 active complex-spike cells (4.9 %) had their firing organized in both spatial frames.

This partial overlap between the room and arena representations is consistent with previous reports of incomplete pattern separation in CA1 [40,41]. During two-frame avoidance this could manifest as a modulation of cell firing by the other frame as the rat’s experience of a particular place changes with the angular displacement of the arena in the room. Further work will be necessary to determine whether partial integration of the two streams of spatial information contributes to the tendency for coordinated concordant discharge within the ensemble and whether this contributes to cognitive control of the two information streams.

*Characteristics of room-preferring and arena-preferring ensemble states.*

Periods of predominant room *I_pos_* and predominant arena *I_pos_* are accompanied by similar speed of the rats’ movement. For each recording speed during the periods of predominant room information and predominant arena information was computed and compared by a t-test. Each recording was analyzed separately. Out of 10 recordings t-test indicated significant difference (p<0.05) in only one case, which is to be expected by chance.

Further analysis showed that periods of predominant room *I_pos_* and predominant arena *I_pos_* are accompanied by similar amount of theta activity. For nine sessions (in one session no EEG was recorded.) the EEG was analyzed and the power spectra were computed for one-second intervals. Power at theta frequencies (4-10 Hz) was compared by 2-way ANOVA, the frequency and *I_pos_* ensemble frame preference were the independent factors. In each recording ANOVA showed an effect of frequency, with peak power between 7 and 9 Hz, indicating a predominance of theta activity. The significant effect of ensemble frame preference was only observed in one of nine recordings, as was the significant interaction. These analyses indicate that the frame preference of ensemble activity is not due to different state in rat’s behavior or overall network EGG activity.

Next we studied the instances of changes between predominantly room ensemble activity and predominantly arena activity. During transitions between the ensemble activity we observed higher speed of the rat’s movement (15.1+0.83 cm/s) compared to speed during randomly chosen intervals (11.7+1.06 cm/s) (paired t-test: t_9_ = 3.12; p = 0.01). The theta power (5-10 Hz) was not different during transitions between the ensemble activity compared to randomly chosen intervals (paired t-test: t_8_ = 1.21; p = 0.26). Neither was the interneuron firing rate different during transitions. However, we only analyzed the activity of six interneurons that were recorded together with the large ensembles of putative pyramidal cells. (paired t-test: t_5_ = 0.42; p = 0.69).

Next we compared the ensemble frame preference during the entire session and frame-specific avoidance behavior. The proportion of room- and arena-preferring cells in the two-frame avoidance sessions was not related to the proportion of entrances to the room shock zone versus the arena shock zone, neither was there a relationship to the session-averaged relative distances from the two shock zones (Figure S10).

*Fear during the two-frame task*

Fear during the active place avoidance task was evaluated previously in unpublished experiments. The animals were placed on a rotating arena and reinforced to avoid only the shock zone defined relative to the room landmarks. Fear during training was evaluated by assessing defecation [42] and compared to fear during exploration of a new environment. Each naïve rat had four 10-min sessions, one per day. The first session was an exploration trial, without shock. The next three sessions were active place avoidance sessions with shock. Defecation during the first active place avoidance session increased compared to the exploration session. With further active place avoidance training defecation decreased to the level during exploration. The effect of session was significant (F_3,63_ = 4.24; p<0.01), and Newman-Keuls post-hoc comparisons confirmed that the first avoidance session was different from the exploration session, as well as the subsequent avoidance sessions (p’s < 0.05). Defecation during the exploration session and the second and third avoidance sessions did not differ significantly.

These results suggest that the rats did not express a fear response during the recordings we studied. Furthermore, the rats did not freeze during the avoidance task; in fact, if they did not move they would not have been able to perform the task. Near continuous locomotion during active place avoidance can be see in Video S1.

*Momentary positional information (I_pos_)*

We aimed to evaluate whether the spatial tuning of place cell discharge changed from one spatial frame to another on short time scales, which is why we sought a measure that estimates how much the discharge during a short time interval signals about the rat’s location at that moment. We began with the measure *I_pos_(x)*, positional information that we introduced in prior work [43],

$I_{pos}\left(x\right)=\sum_{N\geq0} P_{n|x}\log_{2}\left(\frac{P_{n|x}}{P_{n}}\right)$ (1)

*P_n_* is the probability of the cell discharging *n* spikes; *P_n|x_* is the probability of discharging *n* spikes in a short interval of time (~100 ms) if the rat is in location *x*, and the averaging is done with respect to the distribution of all the spike counts *N* that were observed in location *x* throughout the recording. Averaging *I_pos_(x)* across all locations *X* is equal to Shannon’s mutual information.

Although *I_pos_(x)* is always nonnegative the components of the summation can be either positive or negative and the magnitude of the contribution depends on the distinction between the conditional and unconditional probabilities. Consider for example a brief moment when the rat visits a location in the cell’s firing field. Despite being in the firing field the cell may or may not discharge at a high rate [5,6,28]. Robust discharge is more probable in the firing field than on average, so robust discharge will contribute a positive value to the *I_pos_(x)* summation. If on the other hand, the cell fails to discharge, zero spikes is less probable in the firing field than on average, and the moment will contribute a negative value to *I_pos_(x)*. The more the conditional probability of the observed discharge deviates from the unconditional probability, in other words, the more surprising the observation is, the more it contributes to *I_pos_(x)*. In part for this reason, it was suggested that the measure be called “specific surprise” rather than information [44]. They define a different measure they call “specific information” that can have both positive and negative values and argue it should properly be called “specific information” because it has the property of being additive which *I_pos_(x)* does not. Both specific surprise and specific information define Shannon’s mutual information when averaged across all X, which in our case are locations.

Our aim is to characterize the location-specific firing of a cell during a brief time interval (Δt), and our measure is not constrained to average to mutual information, or any other value. We therefore defined momentary positional information – $I_{pos}\left(t\right)$ as a single component of the summation for *I_pos_(x)*:

$I_{pos}\left(t\right)=P_{n|x}\log_{2}\left(\frac{P_{n|x}}{P_{n}}\right)$ (2)

$I_{pos}\left(t\right)$ is zero if the discharge observed in a location has the same unconditional probability (independent of location) as the conditional probability in the current location. $I_{pos}\left(t\right)$ can be positive or negative. However, as described above, the absolute value of $I_{pos}\left(t\right)$ is large whenever the probability of the number of spikes observed at the location is distinct or “surprising” compared to the location-independent, unconditional probability of observing the same number of spikes. We denote |$I_{pos}\left(t\right)$| using the shorthand, *I_pos_*.

We computed the conditional and unconditional probabilities as described in our prior work [43]. Each time step (Δt = 117 ms) we counted the number of spikes a cell emitted. To compute the conditional probability, the spike count histogram at the current location was incremented. The location was estimated as the average position the rat occupied during the time step. To compute the unconditional probability, we incremented the location-independent spike count histogram. Note we do not average the spike counts in these histograms to compute a rate, rather once the histograms are built from the entire recording they define location-specific (conditional) and location-independent (unconditional) probability distributions for the cell.

*Computing I_pos_*

Two adjustments were made for the computation of *I_pos_*. First, we did not attempt to estimate *I_pos_* when the rat visited a location that was sampled for less than one second during the recording session. This was an effort to avoid overestimating *I_pos_* by determining the conditional probabilities from undersampled locations. We point out that this was a conservative choice because we already showed that in practice, undersampled locations do not overestimate positional information [43]. The second adjustment avoided the tautology of using the data from the current time step to estimate the two probabilities for computing *I_pos_*. While the tautology is necessary for computing *I_pos_(x)* so that it averages to mutual information, *I_pos_* is not similarly constrained.

We compared *I_pos_* to established measures of spatial firing quality: spatial coherence [45] and information content (IC), [46]. The properties of these three parameters are illustrated using a database of 162 putative hippocampal pyramidal cells, which were recorded in 16 sessions from 7 rats during place avoidance on the stable arena. The scatter plots in figure S11B and S11C show the distribution of spatial coherence, IC, and *I_pos_* in the database of cells, and the relationships between these measures. The relationship between the three measures and the firing rate of cells is illustrated in figures S11D, E, and F. Figure S11A shows the firing rate maps of seven of the complex-spike cells. A color was assigned to each cell that is indicated by the rectangle around the cell’s firing rate map. The corresponding colors can be used to identify the seven cells in the scatter plots of figure S11B-F. Spatial coherence seems to characterize the firing field quality the best. The cells with high-quality firing fields – green, blue, gray and pink – are also cells with the highest coherence as can be seen in figure S11B. On the other hand, the red and yellow cells have extremely poor firing fields and also the lowest coherence. IC seems to be the worst of the three parameters at estimating spatial firing quality. Although the blue cell has a firing field that appears to be better than the red cell’s, the red cell has higher IC. Similarly, although the green cell has a better firing field, the yellow cell has higher IC. The average *I_pos_* seems to be a useful parameter to characterize spatial firing; its main advantage is the potential to characterize spatial firing during short time intervals.

Figure S11 (D, E, and F) shows how the three parameters are related to the firing rate of a cell. IC is highest for cells with the lowest firing rate. This is not surprising given that IC measures the uniqueness of firing rates in the session-averaged firing rate map. The less the cell fires, the smaller the firing field is, the better we can predict the rat’s position from observing a spike.

**References**

1. Gray CM (1999) The temporal correlation hypothesis of visual feature integration: still alive and well. Neuron 24: 31-47, 111-125.

2. Milner PM (1974) A model for visual shape recognition. Psychol Rev 81: 521-535.

3. von der Malsburg C (1999) The what and why of binding: the modeler's perspective. Neuron 24: 95-104, 111-125.

4. von der Malsburg C (1981) The correlation theory of brain function (MPI Biophysical Chemistry, internal report 81-82). Berlin: Springer, 1994.

5. Fenton AA, Lytton WW, Barry JM, Lenck-Santini P-P, Zinyuk LE, et al. (2010) Attention-like modulation of hippocampus place cell discharge. Journal of Neuroscience in press.

6. Jackson J, Redish AD (2007) Network dynamics of hippocampal cell-assemblies resemble multiple spatial maps within single tasks. Hippocampus 17: 1209-1229.

7. Huxter J, Burgess N, O'Keefe J (2003) Independent rate and temporal coding in hippocampal pyramidal cells. Nature 425: 828-832.

8. Dragoi G, Buzsaki G (2006) Temporal encoding of place sequences by hippocampal cell assemblies. Neuron 50: 145-157.

9. Harris KD, Csicsvari J, Hirase H, Dragoi G, Buzsaki G (2003) Organization of cell assemblies in the hippocampus. Nature 424: 552-556.

10. Diba K, Buzsaki G (2008) Hippocampal network dynamics constrain the time lag between pyramidal cells across modified environments. J Neurosci 28: 13448-13456.

11. Lisman JE (1999) Relating hippocampal circuitry to function: recall of memory sequences by reciprocal dentate-CA3 interactions. Neuron 22: 233-242.

12. Samsonovich A, McNaughton BL (1997) Path integration and cognitive mapping in a continuous attractor neural network model. J Neurosci 17: 5900-5920.

13. Tsodyks M (1999) Attractor neural network models of spatial maps in hippocampus. Hippocampus 9: 481-489.

14. Touretzky D, Muller RU (2006) Place field dissociation and multiple maps in hippocampus Neurocomputing 69: 1260-1263

15. Fenton AA, Kao H-Y, Neymotin SA, Olypher AV, Vayntrub Y, et al. (2008) Unmasking the CA1 ensemble place code by exposures to small and large environments: more place cells and multiple, irregularly-arranged, and expanded place fields in the larger space. J Neurosci 28: 11250-11262.

16. Olypher AV, Klement D, Fenton AA (2006) Cognitive disorganization in hippocampus: a physiological model of the disorganization in psychosis. J Neurosci 26: 158-168.

17. Wesierska M, Dockery C, Fenton AA (2005) Beyond memory, navigation, and inhibition: behavioral evidence for hippocampus-dependent cognitive coordination in the rat. J Neurosci 25: 2413-2419.

18. Cimadevilla JM, Wesierska M, Fenton AA, Bures J (2001) Inactivating one hippocampus impairs avoidance of a stable room-defined place during dissociation of arena cues from room cues by rotation of the arena. Proc Natl Acad Sci U S A 98: 3531-3536.

19. Kubik S, Fenton AA (2005) Behavioral evidence that segregation and representation are dissociable hippocampal functions. J Neurosci 25: 9205-9212.

20. Pastalkova E, Serrano P, Pinkhasova D, Wallace E, Fenton AA, et al. (2006) Storage of spatial information by the maintenance mechanism of LTP. Science 313: 1141-1144.

21. Serrano P, Friedman EL, Kenney J, Taubenfeld SM, Zimmerman JM, et al. (2008) PKMzeta maintains spatial, instrumental, and classically conditioned long-term memories. PLoS Biol 6: 2698-2706.

22. Bannerman DM, Good MA, Butcher SP, Ramsay M, Morris RG (1995) Distinct components of spatial learning revealed by prior training and NMDA receptor blockade. Nature 378: 182-186.

23. Brown JE, Skaggs WE (2002) Concordant and discordant coding of spatial location in populations of hippocampal CA1 pyramidal cells. J Neurophysiol 88: 1605-1613.

24. Shapiro ML, Tanila H, Eichenbaum H (1997) Cues that hippocampal place cells encode: dynamic and hierarchical representation of local and distal stimuli. Hippocampus 7: 624-642.

25. Knierim JJ (2002) Dynamic interactions between local surface cues, distal landmarks, and intrinsic circuitry in hippocampal place cells. J Neurosci 22: 6254-6264.

26. Lee I, Yoganarasimha D, Rao G, Knierim JJ (2004) Comparison of population coherence of place cells in hippocampal subfields CA1 and CA3. Nature 430: 456-459.

27. Fenton AA, Csizmadia G, Muller RU (2000) Conjoint control of hippocampal place cell firing by two visual stimuli. I. The effects of moving the stimuli on firing field positions. J Gen Physiol 116: 191-209.

28. Fenton AA, Muller RU (1998) Place cell discharge is extremely variable during individual passes of the rat through the firing field. Proc Natl Acad Sci U S A 95: 3182-3187.

29. Olypher AV, Lansky P, Fenton AA (2002) Properties of the extra-positional signal in hippocampal place cell discharge derived from the overdispersion in location-specific firing. Neuroscience 111: 553-566.

30. Redish AD, Rosenzweig ES, Bohanick JD, McNaughton BL, Barnes CA (2000) Dynamics of hippocampal ensemble activity realignment: time versus space. J Neurosci 20: 9298-9309.

31. Gothard KM, Skaggs WE, Moore KM, McNaughton BL (1996) Binding of hippocampal CA1 neural activity to multiple reference frames in a landmark-based navigation task. J Neurosci 16: 823-835.

32. Gothard KM, Skaggs WE, McNaughton BL (1996) Dynamics of mismatch correction in the hippocampal ensemble code for space: interaction between path integration and environmental cues. J Neurosci 16: 8027-8040.

33. Rivard B, Li Y, Lenck-Santini PP, Poucet B, Muller RU (2004) Representation of objects in space by two classes of hippocampal pyramidal cells. J Gen Physiol 124: 9-25.

34. Lenck-Santini PP, Rivard B, Muller RU, Poucet B (2005) Study of CA1 place cell activity and exploratory behavior following spatial and nonspatial changes in the environment. Hippocampus 15: 356-369.

35. Hampson RE, Simeral JD, Deadwyler SA (2002) "Keeping on track": firing of hippocampal neurons during delayed-nonmatch-to-sample performance. J Neurosci 22: RC198.

36. Eichenbaum H, Wiener SI, Shapiro ML, Cohen NJ (1989) The organization of spatial coding in the hippocampus: a study of neural ensemble activity. J Neurosci 9: 2764-2775.

37. Redish AD, Battaglia FP, Chawla MK, Ekstrom AD, Gerrard JL, et al. (2001) Independence of firing correlates of anatomically proximate hippocampal pyramidal cells. J Neurosci 21: RC134.

38. Nakamura NH, Fukunaga M, Akama KT, Soga T, Ogawa S, et al. (2010) Hippocampal cells encode places by forming small anatomical clusters. Neuroscience 166: 994-1007.

39. Hampson RE, Simeral JD, Deadwyler SA (1999) Distribution of spatial and nonspatial information in dorsal hippocampus. Nature 402: 610-614.

40. Skaggs WE, McNaughton BL (1998) Spatial firing properties of hippocampal CA1 populations in an environment containing two visually identical regions. J Neurosci 18: 8455-8466.

41. Guzowski JF, Knierim JJ, Moser EI (2004) Ensemble dynamics of hippocampal regions CA3 and CA1. Neuron 44: 581-584.

42. Fanselow MS (1986) Associative vs. topographical accounts of the immediate-shock freezing deficit in rats: Implications for the response selection rules governing species-specific defensive reactions. Learning and Motivation 17: 16–39.

43. Olypher AV, Lansky P, Muller RU, Fenton AA (2003) Quantifying location-specific information in the discharge of rat hippocampal place cells. J Neurosci Methods 127: 123-135.

44. DeWeese MR, Meister M (1999) How to measure the information gained from one symbol. Network 10: 325-340.

45. Kubie JL, Muller RU, Bostock E (1990) Spatial firing properties of hippocampal theta cells. J Neurosci 10: 1110-1123.

46. Skaggs WE, McNaughton BL, Gothard KM, Markus EJ (1993) An information theoretic approach to deciphering the hippocampal code. In: Hanson SJ, Cowan JD, Giles CL, editors. Advances in neural information processing. San Mateo, CA: Morgan Kaufmann Publishers. pp. 1030-1037.

47. Wilson MA, McNaughton BL (1993) Dynamics of the hippocampal ensemble code for space. Science 261: 1055-1058.
