# Supplementary figures and images for: Correction: Dynamic Grouping of Hippocampal Neural Activity During Cognitive Control of Two Spatial Frames
Source: PLoS Biol. 2015 Mar 11;13(3):e1002100. doi: 10.1371/journal.pbio.1002100 (PMC4356546; doi:10.1371/journal.pbio.1002100)

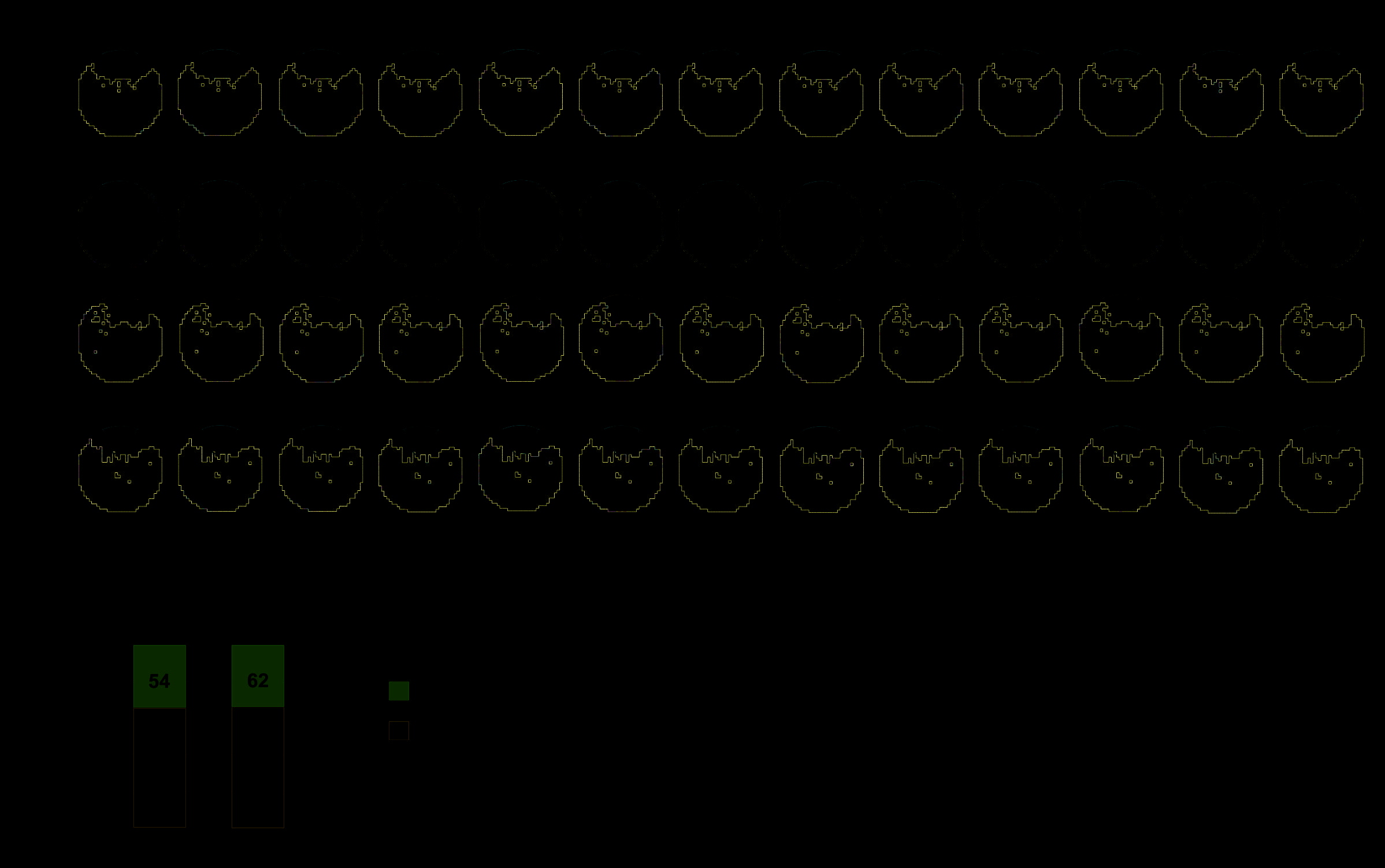

Supplement: S2 Fig — (A) Firing rate maps of 13 cells recorded together during a two-frame avoidance session that was flanked by two sessions of place avoidance on the stationary arena. During rotation the spatial firing of these cells was better organized in the arena frame than in the room frame. Spatial coherence is given below each firing rate map. (B) The proportion of place cells with spatial coherence greater than 0.4 is similar in the stationary and rotating conditions. The 0.4 threshold was chosen because cells with spatial coherence greater than 0.4 are typically considered high quality place cells. (TIF) [file pbio.1002100.s001.tif]
